# Spatiotemporal multi-omics: exploring molecular landscapes in aging and regenerative medicine

**DOI:** 10.1186/s40779-024-00537-4

**Published:** 2024-05-27

**Authors:** Liu-Xi Chu, Wen-Jia Wang, Xin-Pei Gu, Ping Wu, Chen Gao, Quan Zhang, Jia Wu, Da-Wei Jiang, Jun-Qing Huang, Xin-Wang Ying, Jia-Men Shen, Yi Jiang, Li-Hua Luo, Jun-Peng Xu, Yi-Bo Ying, Hao-Man Chen, Ao Fang, Zun-Yong Feng, Shu-Hong An, Xiao-Kun Li, Zhou-Guang Wang

**Affiliations:** 1 Affiliated Cixi Hospital, Wenzhou Medical University, Ningbo, 315300 Zhejiang China; 2 Oujiang Laboratory (Zhejiang Lab for Regenerative Medicine, Vision and Brain Health), School of Pharmaceutical Science, Wenzhou Medical University, Wenzhou, 325035 Zhejiang China; 3 National Key Laboratory of Macromolecular Drug Development and Manufacturing, School of Pharmaceutical Science, Wenzhou Medical University, Wenzhou, 325035 Zhejiang China; 4 State Key Laboratory of Bioelectronics, School of Biological Science & Medical Engineering, Southeast University, Nanjing, 210096 China; 5 School of Pharmaceutical Sciences, Guangdong Provincial Key Laboratory of New Drug Screening, Southern Medical University, Guangzhou, 510515 China; 6 Department of Human Anatomy, Shandong First Medical University & Shandong Academy of Medical Sciences, Taian, 271000 Shandong China; 7 Integrative Muscle Biology Laboratory, Division of Regenerative and Rehabilitative Sciences, University of Tennessee Health Science Center, Memphis, TN 38163 United States; 8 Key Laboratory for Laboratory Medicine, Ministry of Education, Zhejiang Provincial Key Laboratory of Medical Genetics, School of Laboratory Medicine and Life Science, Wenzhou Medical University, Wenzhou, 325035 Zhejiang China; 9 Key Laboratory of Imaging Diagnosis and Minimally Invasive Intervention Research, Institute of Imaging Diagnosis and Minimally Invasive Intervention Research, the Fifth Affiliated Hospital of Wenzhou Medical University, Lishui Hospital of Zhejiang University, Lishui, 323000 Zhejiang China; 10 School and Hospital of Stomatology, Wenzhou Medical University, Wenzhou, 324025 Zhejiang China; 11 Departments of Diagnostic Radiology, Surgery, Chemical and Biomolecular Engineering, and Biomedical Engineering, Yong Loo Lin School of Medicine and College of Design and Engineering, National University of Singapore, Singapore, 119074 Singapore; 12 Clinical Imaging Research Centre, Centre for Translational Medicine, Yong Loo Lin School of Medicine, National University of Singapore, Singapore, 117599 Singapore; 13 Nanomedicine Translational Research Program, NUS Center for Nanomedicine, Yong Loo Lin School of Medicine, National University of Singapore, Singapore, 117597 Singapore; 14 Institute of Molecular and Cell Biology, Agency for Science, Technology, and Research (A*STAR), Singapore, 138673 Singapore

**Keywords:** Spatiotemporal multi-omics, Aging and regeneration, Cellular interactions, Innovative therapeutic strategies

## Abstract

Aging and regeneration represent complex biological phenomena that have long captivated the scientific community. To fully comprehend these processes, it is essential to investigate molecular dynamics through a lens that encompasses both spatial and temporal dimensions. Conventional omics methodologies, such as genomics and transcriptomics, have been instrumental in identifying critical molecular facets of aging and regeneration. However, these methods are somewhat limited, constrained by their spatial resolution and their lack of capacity to dynamically represent tissue alterations. The advent of emerging spatiotemporal multi-omics approaches, encompassing transcriptomics, proteomics, metabolomics, and epigenomics, furnishes comprehensive insights into these intricate molecular dynamics. These sophisticated techniques facilitate accurate delineation of molecular patterns across an array of cells, tissues, and organs, thereby offering an in-depth understanding of the fundamental mechanisms at play. This review meticulously examines the significance of spatiotemporal multi-omics in the realms of aging and regeneration research. It underscores how these methodologies augment our comprehension of molecular dynamics, cellular interactions, and signaling pathways. Initially, the review delineates the foundational principles underpinning these methods, followed by an evaluation of their recent applications within the field. The review ultimately concludes by addressing the prevailing challenges and projecting future advancements in the field. Indubitably, spatiotemporal multi-omics are instrumental in deciphering the complexities inherent in aging and regeneration, thus charting a course toward potential therapeutic innovations.

## Background

**Fig. 1:**
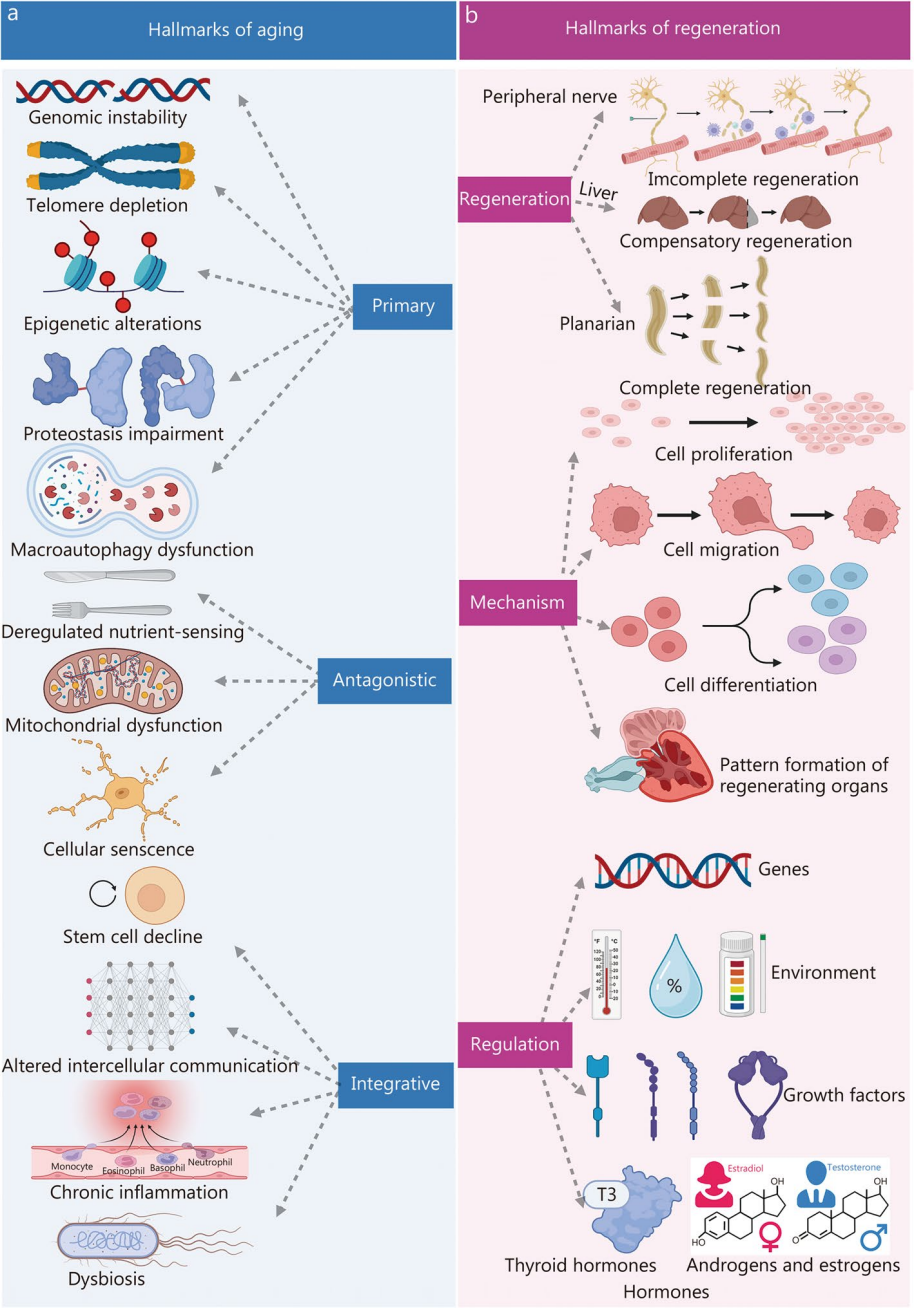
Key features of aging and regenerative processes. **a** The twelve hallmarks of aging included genomic instability, telomere depletion, epigenetic alterations, proteostasis impairment, macroautophagy dysfunction, dysregulated nutrient-sensing, mitochondrial dysfunction, cellular senescence, stem cell decline, altered intercellular communication, chronic inflammation, and dysbiosis. Reprinted with permission from [3]. Copyright © 2022 Elsevier Inc. **b** The hallmarks of regeneration can be classified into three main areas: the types of regeneration, the underlying mechanisms governing it, and the regulatory processes orchestrating regenerative events. Regeneration is divided into three types: complete, incomplete, or compensatory, depending on the extent of restoration achieved. Processes involve cell activities like proliferation, migration, differentiation, and pattern formation. Proliferation creates new tissues through cell division. Migration moves cells for correct structure. Differentiation changes cells into specialized types. Pattern formation arranges new tissues or organs. Genes, environment, hormones, and growth factors affect regeneration, T3 triiodothyronine

Aging and regeneration, are fundamental biological processes, that have long captivated the scientific community. Aging, a multifaceted phenomenon, is characterized by a progressive decline in physiological functions coupled with an increased vulnerability to age-related diseases [1–5]. To elucidate this complex process, López-Otín et al. [3] introduced a comprehensive framework encompassing twelve hallmarks of aging. These hallmarks, which span a range of molecular, cellular, and organ processes, contribute significantly to aging. They include genomic instability, telomere depletion, epigenetic alterations, proteostasis impairment, macroautophagy dysfunction, dysregulated nutrient-sensing, mitochondrial dysfunction, cellular senescence, stem cell decline, altered intercellular communication, chronic inflammation, and dysbiosis (Fig. 1a [3]). These hallmarks furnish pivotal insights into the mechanisms underlying aging and hole potential for informing interventions aimed at promoting healthier aging trajectories and mitigating age-related diseases.

In contrast, regeneration signifies the extraordinary ability of certain organisms to repair and restore damaged or lost cells, tissues, or organs, ultimately achieving tissue homeostasis and functional recovery [6–10]. This process is intricately orchestrated, involving a coordinated cascade of molecular events. The phenomena of aging and regeneration are closely intertwined; notably, a decline in regenerative capacity is frequently associated with aging [11–13]. Deciphering the molecular underpinnings of aging and regeneration is crucial for the development of interventions and therapies that support healthy aging and enhance tissue repair capabilities.

A comprehensive understanding of aging and regeneration necessitates an analysis of molecular dynamics across both spatial and temporal dimensions. The functional state of senescent and those undergoing regenerative repair is intricately controlled by the spatiotemporal regulation of gene expression [14–16]. However, conventional transcriptomics methods fall short of capturing the simultaneous spatial and temporal dependencies of RNA profiles [17–19]. While existing transcriptomics techniques adeptly dissect gene expression in heterogeneous cell types within a histomorphological context, providing static snapshots, they are limited [20–22]. Cutting-edge single-cell sequencing technologies and metabolic RNA labeling methods allow for temporal analysis of nascent single-cell transcriptomes, yet they lack precise spatial resolution [23–25]. Although live cell imaging facilitates the tracking of intracellular RNA trajectories, visualizing multiple transcripts simultaneously remains a significant challenge [26–28]. Thus, the pursuit of sequencing methodologies that combine high multiplexing capabilities with simultaneous spatiotemporal resolution is imperative. Such methodologies would enable in situ monitoring of de novo messenger RNA (mRNA) at subcellular and single-cell resolution. Furthermore, in response to stress signals from the microenvironment, senescent and regenerative cells rapidly assemble functional protein complexes to propagate these signals [29–31]. These cellular signal processes, encompassing post-translational protein modifications, the assembly of functional protein complexes, and the intricate interplay of subcellular spatial migration, contribute significantly to the orchestration and regulation of dynamic protein complexes in a spatiotemporally dynamic manner.

To effectively address the challenges inherent in studying aging and regeneration, the deployment of spatiotemporal multi-omics technology is paramount. Enhanced by the addition of a temporal dimension, spatiotemporal multi-omics facilitates a thorough examination of the temporal variations exhibited by entities such as cells, genes, and proteins during the aging and regeneration processes. These methodologies are instrumental in identifying key regulatory moments and dynamic mechanisms, offering a more intricate portrayal of the temporal characteristics underpinning biological processes and the formation of precise biomolecular networks. Notably, spatiotemporal multi-omics reveal the temporal correlations and interactions that govern molecular dynamics within complex networks throughout the progression of aging and regeneration. Additionally, it unveils insights into the dynamic trajectory of diseases; comparisons of spatiotemporal multi-omics data from healthy and diseased states illuminate the dynamic changes associated with aging and regeneration, providing more accurate insights into the mechanisms driving these processes.

This review endeavors to provide a comprehensive exploration of the application of spatiotemporal multi-omics in the fields of aging and regeneration. It delves into the capabilities of these technologies to probe molecular dynamics, cellular interactions, and signaling pathways within a spatial and temporal framework, thereby shedding light on the multifaceted aspects of these processes. The review commences with an overview of the various spatiotemporal multi-omics methodologies, followed by a detailed examination of recent advancements in the field of aging and regeneration. Importantly, this analysis extends to identifying challenges and offering insights into the future trajectory of these methodologies. Consequently, the integration of spatiotemporal multi-omics approaches emerges as a transformative avenue for untangling the intricacies inherent to aging and regeneration. This, in turn, fosters a fertile ground for the cultivation of innovative therapeutic strategies poised to shape the landscape of future medical interventions.

## Spatiotemporal multi-omics analysis

**Fig. 2:**
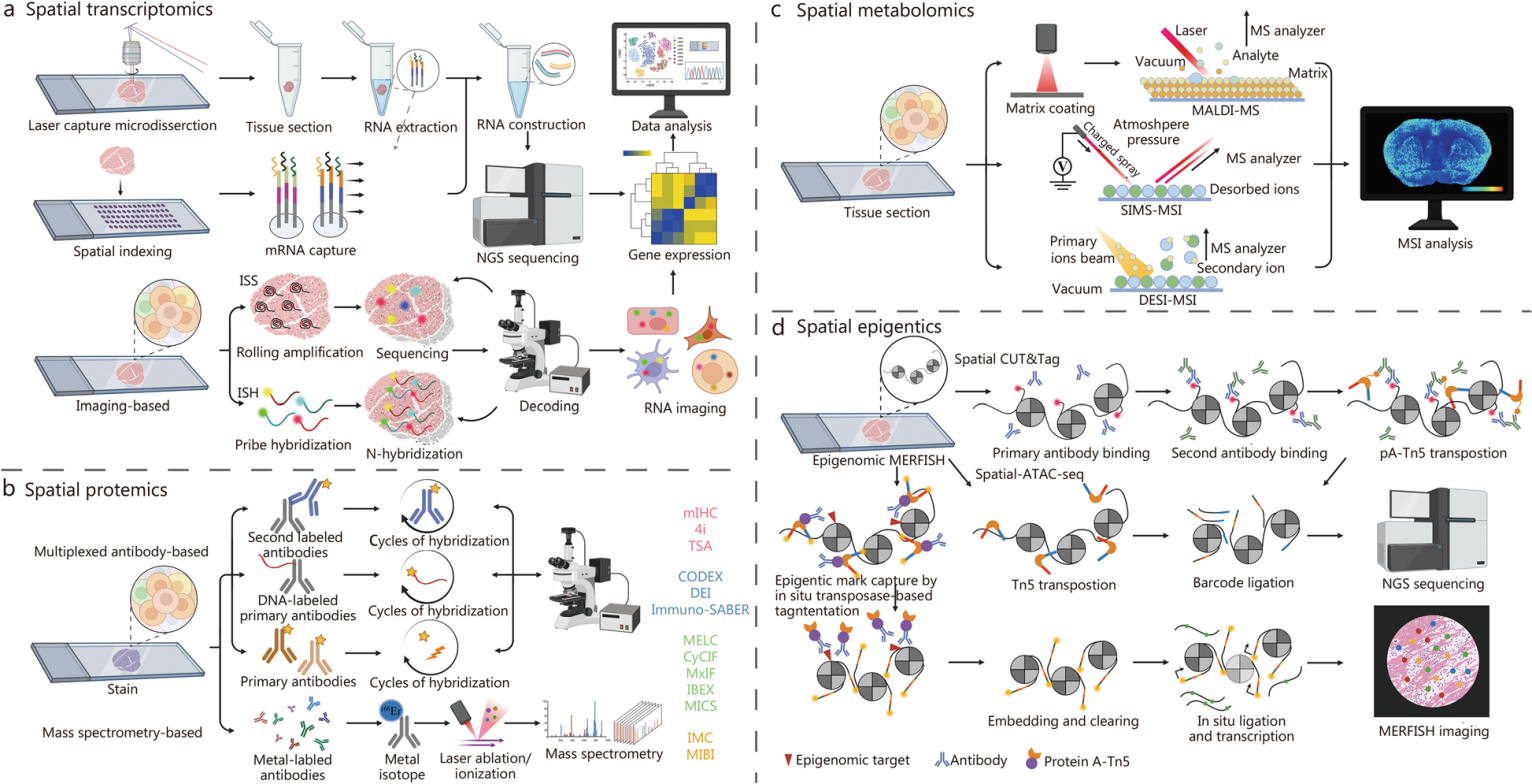
The multi-dimensional approach of spatial multi-omics. **a** Spatial transcriptomics. **b** Spatial proteomics. **c** Spatial metabolomics. **d** Spatial epigenomics. ISS in situ sequencing, ISH in situ hybridization, TSA tyramide signal amplification, MICS MACSima imaging cyclic staining, CODEX co-detection by IndEXing, IMC imaging mass cytometry, MIBI multiplex ionbeam imaging, DESI desorption electrospray ionization, SIMS secondary ion mass spectrometry, MALDI-MS matrix-assisted laser desorption/ionization mass spectrometry, NGS next-generation sequencing, MERFISH multiplexed error-robust fluorescence in situ hybridization, CUT&Tag cleavage under targets and tagmentation, ATAC-seq assay for transposase-accessible chromatin sequencing, mIHC multiplex immunohistochemistry, DEI diffraction enhanced imaging, MELC multi-epitope-ligand cartography, CyCIF cyclic immunofluorescence, MxIF multiplexed immunofluorescence, IBEX Iterative bleaching extends multiplexity, MSI mass spectrometry imaging. 4i iterative indirect immunofluorescence, mIHC multiplex immunohistochemistry, Immuno-SABER immuno-signal amplification by exchange reaction

This section provides a comprehensive overview of the principles, procedures, and categorizations of spatiotemporal multi-omics, which includes spatiotemporal transcriptomics (STT), spatiotemporal proteomics (STP), spatiotemporal metabolomics (STM), and spatiotemporal epigenomics (STE). The introduction previously in this paper establishes a foundational understanding of complex organizational structures on a grander scale and across more elevated dimensions. Spatiotemporal multi-omics can be broadly classified into two principal categories. The first category involves pseudo-temporal analysis, which leverages spatial multi-omics technologies (Fig. 2). This approach entails the systematic collection of tissue samples across a series of temporal stages, followed by meticulous analysis at multiple, distinct time points. Such a method enables a nuanced comprehension of both temporal and spatial fluctuations within the tissue matrix. The second category involves the application of authentic spatiotemporal techniques, which require the labeling of specific intracellular molecules. Subsequent longitudinal tracking allows for the observation and characterization of these molecules' dynamic trajectories through time and space. These approaches collectively enhance our understanding of cellular behavior in complex biological systems. Crucially, these innovative methodologies hold immense potential in unraveling the intricate details underlying the processes of aging and regeneration, thereby driving forward our understanding and fostering the development of groundbreaking therapeutic strategies.

### Overview of STT

The widespread application of single-cell RNA sequencing (scRNA-seq) technology has yielded valuable insights into the diversity of cellular composition and gene expression status in tissues, enabling the resolution of temporal changes through sampling at distinct time points [23–25, 32–36]. It is crucial to recognize that gene expression exhibits both temporal and spatial heterogeneity. The process of scRNA-seq often involves enzymatic digestion or mechanical separation of cells into suspensions before sequencing. This procedure results in the loss of original location information and disrupts the cellular communication network, thus hindering our understanding of cellular interactions with neighboring cells and extracellular matrix. To overcome these limitations, the advent of STT technology emerged as a promising solution. STT aims to provide gene expression profiles while preserving spatial context and concurrently offering data on the temporal dimension, enabling a more comprehensive exploration of cellular interactions within the tissue microenvironment [37–40].

**Fig. 3:**
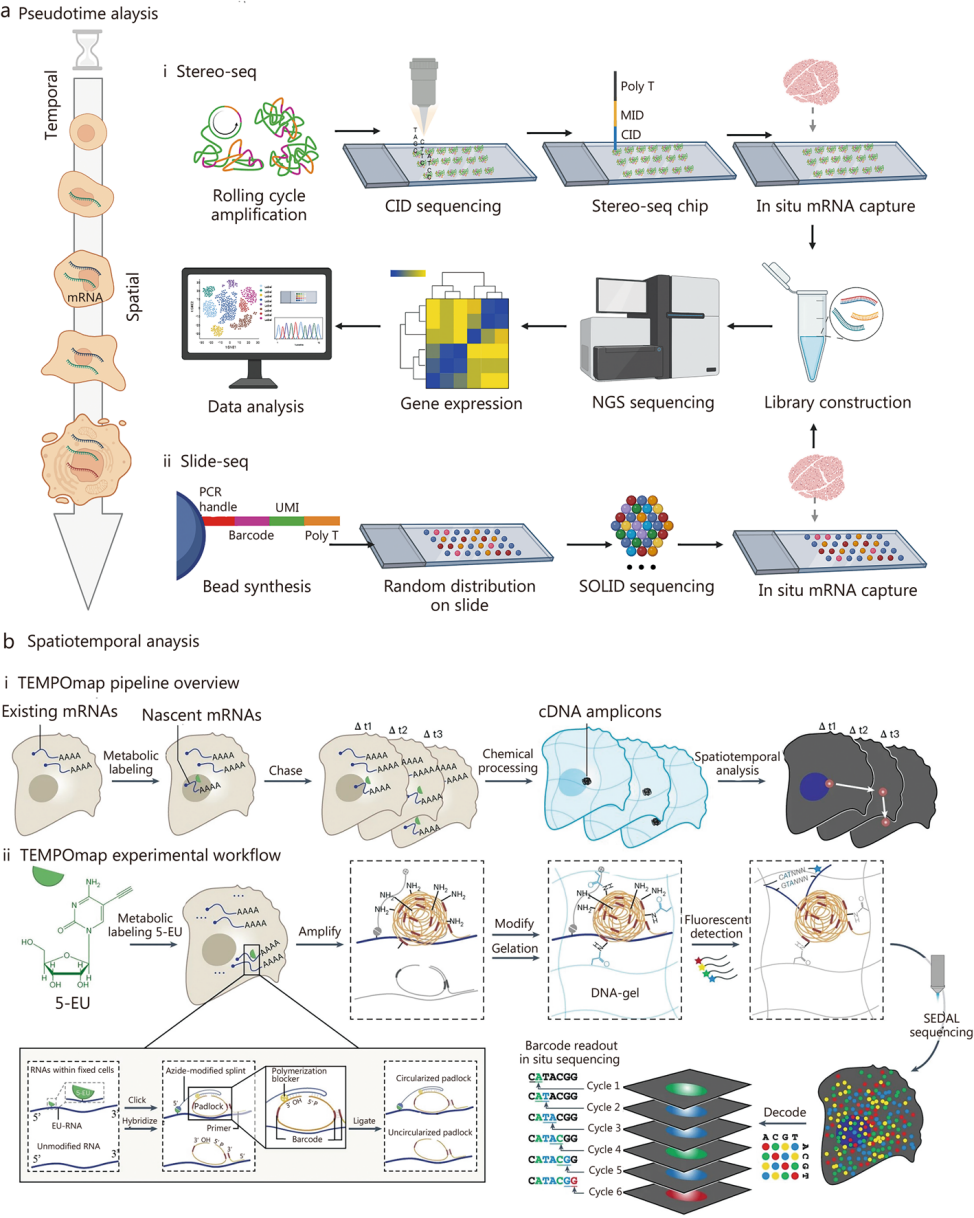
Integrating pseudotemporal analysis with spatiotemporal transcriptomics. **a** Spatial transcriptomic analyses at different time points. (i) Workflows of Sereo-seq. Reprinted with permission from [42]. Copyright © 2022. Published by Elsevier Inc. (ii) Workflows of Slide-seq. Reprinted with permission from [41]. Copyright © 2019, The American Association for the Advancement of Science. **b** Spatiotemporal transcriptomic analysis using 5-ethynyl uridine (5-EU) metabolic markers. (i) TEMPOmap pipeline overview: the process involves collecting and in situ sequencing nascent RNAs from various time points, followed by comprehensive spatiotemporal RNA analysis. (ii) TEMPOmap experimental workflow: the procedure starts with labeling cells using 5-EU. Three distinct probes - splint, primer, and padlock - are combined with cellular mRNAs, leading to enzymatic cDNA amplicon generation from each padlock sequence. These amplicons are integrated into a hydrogel network, secured by a specially designed acrylic component (depicted in blue). The resulting composite structure of DNA and hydrogel is visualized as blue undulating lines. Each amplicon, carrying a unique five-base barcode, undergoes sequential decoding through a six-stage process known as SEDAL fluorescence. This multiplexed RNA quantification approach precisely reveals gene expression patterns in nascent subcellular locations. Reprinted with permission from [43]. Copyright © 2023, Published by Springer Nature. CID coordinate encoding, MID molecularly encoded, NGS next-generation sequencing, PCR polymerase chain reaction, SOLID sequencing by oligonucleotide ligation and detection, SEDAL sequencing with error-reduction by dynamic annealing and ligation, cDNA complementary DNA, UMI unique molecular identifier

STT approaches, developed in recent years, provide a more extensive exploration of the temporal and spatial dimensions of cellular interactions within tissues. These can be broadly divided into two categories. The primary methodology employs a pseudotemporal analysis based on spatial transcriptomics techniques. It involves the systematic collection of tissue sections across various temporal stages. By conducting detailed analyses of these sections at multiple discrete time points, the method elucidates spatiotemporal variations within the tissue matrix. Presently, this pseudotemporal approach is widely applied. Conversely, the secondary methodology employs an authentic STT technique. This approach necessitates the labeling of specific intracellular substances, followed by longitudinal tracking to discern the dynamic spatiotemporal shifts they undergo. A detailed exposition of these two methodologies is presented in the subsequent sections (Fig. 3 [41–43]).

#### Pseudotemporal-based STT

We begin by presenting a brief overview of spatial transcriptomics (Fig. 2a), given that pseudotemporal analyses are fundamentally reliant on these techniques. Spatial transcriptomics technologies can be categorized into two types based on sequencing throughput. The first category includes low-throughput spatial technologies such as microdissected gene expression technologies [44–47], in situ hybridization technologies [48–50], and in situ sequencing methods [51–53]. The second category encompasses high-throughput spatial technologies that utilize spatial barcodes. Examples include Nanostring [54, 55], 10× Visium [56, 57], Slide-seq [41, 58], Slide-seq V2 [57, 59], Stereo-seq [42, 60, 61], Seq-scope [62, 63], and sci-Space [64, 65]. The foundational principles and comprehensive categorization of these techniques have been extensively discussed in our recent publications [66] and elsewhere [44, 47, 67–71]. The primary principles and steps are succinctly summarized (Fig. 3a [41, 42]**)**. This section focuses on providing an overview of high-throughput spatial technologies, with a particular emphasis on Stereo-seq [42, 61, 72–74]. Random barcode sequences are incorporated into DNA nanoballs (DNBs) which are then positioned on a modified chip using photolithographic etching techniques. Subsequently, the array undergoes micro-graphically treatment with primers and sequencing, revealing the sequence of each etched DNB. A data matrix containing the coordinate encoding (CID) for each etched DNB is obtained. Molecularly encoded (MID) and oligonucleotide-containing polyp sequences are then bound to each position through hybridization with the CID. Frozen sections of fresh tissue are placed onto the chip’s surface for fixation, permeabilization, reverse transcription, and amplification, aimed at capturing polyA-tailed RNA from the tissue. The amplified complementary DNAs (cDNAs) serve as templates for library preparation and co-sequenced with CID. In standard data analysis, the 1 cm × 1 cm chip incorporates 400 million DNBs, a result of amplifying [61]. The Stereo-seq technology overcomes the limitations of previous spatial transcriptomic methods by simultaneously enhancing resolution, gene capture efficiency, and field of view. By introducing a novel combination of DNB patterning and in situ, RNA capture, Stereo-seq establishes a new standard for generating high-resolution, comprehensive STT atlases, holding promise for application in the realms of aging and regeneration research. It is expected to uncover potential regulatory mechanisms underlying age-related changes and tissue regeneration, thus advancing our understanding of aging and regeneration. Moreover, this technology will offer a framework for targeted interventions in age-related diseases and regenerative medicine.

#### Spatiotemporal-based STT

The authentic STT involves the tagging of individual cells or distinct intracellular components to observe their spatiotemporal dynamics. Presently, metabolic labeling of mRNA is instrumental in distinguishing newly synthesized mRNA molecules from those synthesized earlier. Due to advancements in biochemical methodologies, a range of RNA metabolic labeling techniques have been integrated into scRNA-seq. These methods enable the discrimination between “old” and “new” RNAs [75]. Commonly, modified exogenous nucleotides, such as 4-thiouridine (s^4^U) [76] and 5-ethynyl uracil [77], are employed for labeling newly transcribed RNAs. Through oxidative nucleophilic substitution, these exogenous nucleotides can be converted into different nucleotides, introducing base mutations in cDNA. This process facilitates the differentiation of “old” and “new” RNAs based on mutation sites in subsequent sequencing. Integrating the RNA metabolic labeling with high-throughput scRNA-seq, Qiu et al. [78] developed metabolic labeling-based single-cell RNA tagging sequencing (scNT-seq) using droplet microfluidic technology. In this technique, cells labeled with s^4^U are encapsulated in droplets along with barcoded beads. Following cell lysis, the beads capture both pre-existing and s^4^U-labeled newly transcribed RNA. The s^4^U on the beads is then chemically converted to cytosine derivatives, allowing base-pair with guanine during transcription. This process enables the location and information of new transcripts to be inferred from sequencing reads showing T to C substitutions. Although scNT-seq provides high-throughput temporal information on cellular mRNAs, it does not offer detailed transcriptional dynamics on an hourly scale.

To enhance understanding of the temporal dynamics in gene expression, Rodriques et al. [79] developed an innovative RNA timestamping technique. This method is designed to ascertain the “age” of individual RNA molecules and monitor historical shifts in gene expression within single cells. Utilizing an adenine-rich RNA template with an MS2 binding site, this RNA timestamp serves as a substrate for the enzyme adenine deaminase ADAR. In this methodology, the catalytic domain of ADAR is fused with the MS2 capsid protein. This fusion allows ADAR to specifically target and edit adenine within the MS2 binding site, leading to adenine-to-inosine (A-to-I) mutations. As ADAR binds to the RNA timestamps over time, these A-to-I edits accumulate, providing a reliable measure of the age of RNA molecules on an hourly basis. Additionally, a molecular biology protocol integrating this timestamping technique with droplet-based RNA sequencing was developed. This combination demonstrates the compatibility of the timestamping system with advanced, high-throughput single-cell transcriptomic sequencing methods. Applying this methodology enables the discernment of transcriptional changes at specific time intervals, revealing cellular diversity and applying these insights to various biological systems. This approach not only enhances understanding of transcriptional processes but also holds potential for tracking stimulus-specific responses. However, current metabolic RNA labeling methods, while enabling temporal analysis of nascent single-cell transcriptomes, lack essential spatial resolution. Although live cell imaging can track intracellular RNA trajectories, visualizing multiple transcripts concurrently remains a formidable challenge [80, 81]. There is a pressing need for highly multiplexed sequencing techniques that offer both spatial and temporal resolution, capable of effectively tracing nascent mRNA from its inception to its conclusion at subcellular and single-cell levels.

In response to this need, Ren et al. [43] developed the TEMPOmap method, designed for tracing the spatiotemporal progression of the nascent transcriptome at subcellular resolution. TEMPOmap employs the selective amplification of metabolic-labeled and pulse-labeled nascent transcriptomes, combined with advanced three-dimensional (3D) in situ RNA sequencing within hydrogel cellular scaffolds. The introduction of pulse-tracking markers enables simultaneous tracking of multiple genes throughout their RNA lifecycle, capturing vital kinetic parameters such as transcription, decay, nuclear export, and cytoplasmic translocation rates. Analysis of these spatiotemporal metrics reveals that different mRNAs undergo distinct regulation at various stages of the RNA life cycle and during cell cycle phases, influencing gene functionality. TEMPOmap represents a novel method for spatiotemporally resolved transcriptomics, utilizing metabolically labeled RNA along with a triad of probe sets: a splint DNA probe, a padlock probe, and a primer-probe. The splint DNA probe establishes a covalent bond with labeled mRNA via copper(I)-catalyzed azide-alkyne cycloaddition. Simultaneously, the padlock probe identifies the mRNA target and can loop when adjacent to the splint DNA probe on the same RNA molecule. In situ, primer probes amplify these circular padlocks through rolling circular amplification, leading to the formation of cDNA nanospheres or amplicons. This amplification occurs only for mRNAs that simultaneously bind all three probes, ensuring selective detection of labeled mRNA populations. To facilitate highly multiplexed transcriptome detection, in situ-generated cDNA amplicon libraries were embedded within hydrogel matrices. This was followed by several rounds of fluorescence imaging. Subsequently, the genes encoded by barcodes were deciphered using sequencing for error reduction by dynamic annealing and ligation (SEDAL). This approach was thoroughly tested on human cells, encompassing 991 genes, which displayed diverse spatial and temporal RNA expression patterns (Fig. 3b [43]).

As a key exemplar in the realm of spatiotemporal transcriptome research, TEMPOmap stands out for its innovative approach. This advanced technology in single-cell transcriptomics enables the simultaneous analysis of RNA with remarkable subcellular and temporal precision. It proficiently demonstrates TEMPOmap’s capability to trace the subcellular distribution and cytoplasmic translocation of transcripts over time, thereby providing a comprehensive insight into RNA subcellular dynamics at the single-cell level. Moreover, the observed strong correlation between the dynamic patterns of RNA and the molecular function of genes suggests that a functionally driven regulation of the RNA lifecycle has likely evolved to manage spatiotemporal gene expression with both precision and efficiency [82]. The validation of TEMPOmap was conducted across various cell types, including human induced pluripotent stem cell-derived cell cultures and primary cell cultures. This process illuminated the cell type-specific regulation of RNA dynamics [43]. While the kinetics of RNA generally mirror the inherent characteristics of genes grouped by molecular function, the kinetic behavior of genes crucial to specific cellular functions is notably influenced by the cell’s state and type. It is important to recognize that TEMPOmap may display sequence bias and necessitates the use of uridine analogs for metabolic labeling and the formulation of DNA probes.

### An insight into STP

Traditional proteomic analyses of bulk tissues often fail to the spatial distribution and cellular specificity due to tissue homogenization, resulting in merely an average representation of protein expression levels in the mass spectrometry signal [82]. Conversely, advanced spatial proteomics provides a precise delineation of protein expression profiles across varied cells and tissue regions [83]. The integration of spatial, cell-type, and proteomic data offers critical insights into tissue spatial microenvironments. The integration facilitates the identification of accurate biomarkers and elucidates novel functional pathways [84]. While spatial transcriptomics effectively captures spatial data through methods like multiplexed fluorescence in situ hybridization or sequencing, inferring protein expression from transcriptomic data is a challenging endeavor [17]. The relationship between mRNA and its resulting protein is intricate and non-linear, often leading to discrepancies between mRNA and protein expression levels [85, 86]. Notably, transcript expression tends to exhibit more variability compared to protein expression, with proteins generally presenting lower variation coefficients relative to their corresponding mRNAs [87]. Direct spatial proteomic measurements thus provide a more authentic depiction of cellular functions and states.

**Fig. 4:**
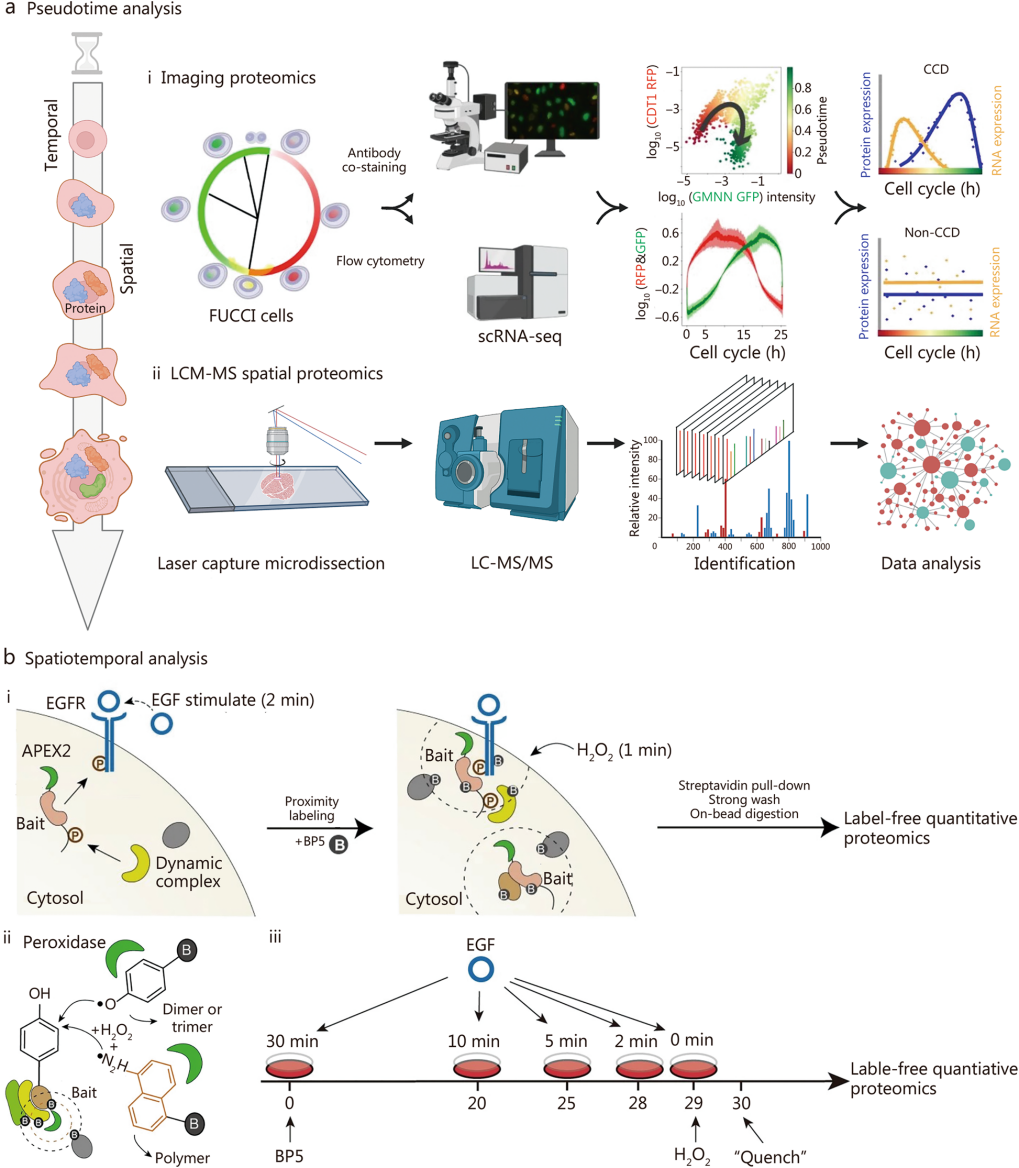
Temporal pseudotemporal analysis and spatiotemporal proteomic analysis. **a** Spatial proteomics analyses at different time points. (i) Spatial proteomic analysis uses U2OS FUCCI cells with dual fluorescent cell cycle indicators (CDT1 in G1 marked by red RFP and GMNN in S and G_2_ highlighted in green GFP). This system provides insights into cell cycle behavior, particularly during the G_1_ – S transition, where both markers are active, creating a distinct yellow hue. Using a polar model for RFP and GFP intensity, the cell cycle’s progression is streamlined into a linear format, facilitating the comparison of independent RNA and protein expression measurements aligned by individual cell pseudotime. Reprinted with permission from [91]. Copyright © 2021, Published by Springer Nature. (ii) LCM-MS spatial proteomics: utilizing laser capture microdissection followed by mass spectrometry (LCM-MS) for spatial proteomic analysis. **b** Spatiotemporal proteomics based on proximity labeling. (i) BP5 proximity proteomics: studies the adapter protein interactome response to epidermal growth factor (EGF) stimulation in living cells using BP5-based proximity labeling. (ii) Peroxidase-catalyzed proximity labeling: uses peroxidases for proximity labeling in both living cells and in vitro settings. (iii) Proximity proteomics workflow: involves a five-time-course EGF stimulation in HeLa cell lines stably expressing APEX2-FLAG-STS1. After EGF stimulation, cells are labeled with BP5 as indicated in the study design. Reprinted with permission from [90]). Copyright © 2021, Published by Springer Nature. CCD cell-cycle-dependent, scRNA-seq single-cell RNA sequencing, CDT1 chromatin licensing and DNA replication factor 1, RFP red fluorescent protein, GMNN geminin, GFP green fluorescent protein, EGFR epidermal growth factor receptor

STP unveils unparalleled perspectives on protein dynamics, localization, and interactions across both spatial and temporal dimensions. It offers deeper insights into cellular processes than what can be gleaned from spatial or bulk proteomics alone [88, 89]. STP can be categorized into two primary methodologies, each characterized by its distinct approach to in vivo protein labeling for tracking intricate spatial and temporal dynamics (Fig. 4 [90, 91]). The first approach utilizes pseudotemporal analysis, where spatial proteomics techniques are employed to meticulously examine tissue sections at defined temporal intervals. Fortified by algorithms specifically designed for pseudotemporal analysis, this method extrapolates protein behaviors across the temporal and spatial spectra. Conversely, the second methodology is rooted in genuine STP. This sophisticated approach involves the in vivo tagging of targeted proteins localized within specific subcellular organelles, utilizing biotin in a live cell environment. The overarching objective here is to astutely monitor and decipher the nuanced alterations in these proteins across the intertwined dimensions of space and time [90]. While the basics of pseudotemporal analysis are well-established in spatial proteomics literature, our primary focus in this discourse is on the latter, the authentic aspect of STP. An in-depth overview of spatial proteomic methodologies is essential before delving into STP, as STP is intrinsically built upon the foundational concepts and techniques of spatial proteomics.

#### Spatial proteomics

Spatial proteomics, an advanced field of study, utilizes a range of techniques, such as immunohistochemistry, immunofluorescence, mass spectrometry, and cytometry, to delineate protein distributions across scales ranging from whole tissues to subcellular entities [92, 93]. Navigating these techniques involves careful consideration of trade-offs, including spatial resolution, analytical depth, molecular and cellular throughput, as well as data acquisition durations [94, 95]. The techniques can be broadly categorized into two main groups: multiplexed antibody-based approaches and mass spectrometry-based methods, depending on the use of antibodies or other specific conjugates (Fig. 2b and Table 1 [96–114]).

**Table 1:** Spatial proteomics platforms

**Type**	**Characteristics**	**Resolution**	**Tissue preparation**	**References**
Fluorescence-based methods	Involves immunostaining, fluorescence image acquisition, and inactivation/removal processes; Techniques: SWITCH, MxIF, t-CyCIF, IBEX, 4i, and OPAL	Single-cell to sub-cellular	FFPE and FF	[96–102]
Metal-based methods	Uses metal-coupled antibodies; Detection through mass spectrometry; IMC uses a single laser; MIBI-TOF uses two source ion beams	Single-cell	FFPE and FF	[103–105]
DNA barcoded-methods	Overcomes spectral constraints of fluorescence; Techniques include DEI, CODEX, and immuno-SABER	Single-cell	FFPE and FF	[106–109]
Matrix-assisted laser desorption/ionization (MALDI)	A pulsed laser beam ionizes primarily small biomolecules and peptides; Used in investigating diseases, including cancer	Near single-cell	FFPE and FF	[110, 111]
Deep visual proteomics (DVP)	Fuses AI-guided image-based segmentation with ultra-high sensitivity MS-based proteomics, linking visual cues to potential proteomic attributes	Sub-cellular	FFPE and FF	[112, 113]
Expansion proteomics (ProteomEx)	Combines tissue expansion via hydrogel embedding with MS-based proteomics to identify disease-associated proteins in specific tissue subregions	About 160 µm	Small volumes (0.61 nl)	[114]

*SWITCH* a simple method that enables proteomic imaging for scalable, integrated, high-dimensional phenotyping of both animal tissues and human clinical sample, *MxIF* multiplex immunofluorescence, *t-CyCIF* tissue-based circular immunofluorescence, *IBEX* iterative bleach extended multiplexing, *4i* iterative indirect immunofluorescence, *SABER* signal amplification by exchange reaction, *DEI* diffraction enhanced imaging, *CODEX* co-detection by indexing, *FFPE* formalin-fixed paraffin-embedded, *FF* fresh frozen, *IMC* imaging mass cytometry, *MIBI-TOF* multiplexed ion beam imaging by time of flight, *AI* artificial intelligence, *MS* mass spectrometry

Multiplexed antibody-based techniques employ various antibody labeling mechanisms, including fluorophores, metal markers, and DNA barcodes [94]. Each labeling method has its advantages and disadvantages, necessitating the use of validated antibodies for accuracy. Notable fluorescent techniques include SWITCH [96], multiplex immunofluorescence [97], tissue-based circular immunofluorescence [98, 99], and iterative bleach extended multiplexing [100], each distinguished by its approach to fluorescence signal removal. Indirect methods include the iterative indirect immunofluorescence [101] and the OPAL system [102]. Metal-based imaging methods, such as imaging cytometry [103, 104] and multiplex ion beam imaging by time of flight [105], utilize metal-coupled antibodies and mass spectrometry for detection. DNA barcoded techniques like DNA exchange imaging [106], CODEX [107, 108], and immuno-SABER [109], address the spectral limitations of fluorescence and bolster multiplexing capabilities. Despite the efficiency and versatility of DNA barcoding, meticulous antibody validation remains imperative.

The mass spectrometry-based method offers an alternative to antibody-based techniques. In the matrix-assisted laser desorption/ionization (MALDI) method, a pulsed laser ionizes biomolecules and peptides with near single-cell resolution (10 – 50 μm) [110]. Initially, surface proteins undergo in situ digestion to provide a peptide proteome representation. While MALDI offers high-resolution analysis of biological molecules and clarifies molecular pathways in aging and regeneration, it faces challenges such as sample degradation and matrix interferences [111]. Integrating antibody-based imaging with proteome characterization promises deeper tissue biology insights. The deep visual proteomics concept fuses AI-guided imaging with ultra-sensitive mass spectrometry-based proteomics, enhancing cell phenotype identification [112, 113]. Additionally, expansion proteomics (ProteomEx) enables high-resolution proteome profiling, recently identifying proteins in Alzheimer’s disease (AD)-afflicted mouse brains [114], marking advancements in spatial proteomics.

#### Pseudotemporal-based STP

The initial pseudotemporal analysis presented herein focuses on instances of single-cell proteomics methodologies applied across various phases of the cell cycle. Cell division is intricately controlled by specific proteins, encompassing their presence, and activity. These proteins are meticulously regulated in both temporal and spatial dimensions through mechanisms such as transcriptional regulation, post-translational modifications, and protein degradation [115, 116]. While traditional studies of the cell cycle have concentrated on cell populations, a deeper understanding of the intricate interplay between the cell cycle, senescence, and regeneration is of paramount importance [117]. With the progression of aging, there is an increasing dysregulation of the cell cycle, leading to reduced cellular senescence and regenerative capabilities [118]. This imbalance can precipitate age-related tissue dysfunction and the onset of diseases [119]. Comprehending and effectively manipulating the cell cycle within the contexts of senescence and regeneration are imperative for developing interventions that enhance tissue repair and delay the adverse effects of aging. Consequently, the cell cycle stands as a pivotal nexus linking the domains of aging and tissue regeneration. Previous studies have notably enhanced our comprehension of the cell cycle [115, 116], but technical constraints have limited investigations into the variability of protein expression at the single-cell level. The advent of single-cell analyses has opened novel avenues for cell cycle research. Mahdessian et al. [91] employed single-cell proteomics, coupled with single-cell transcriptomics, to identify 1180 proteins expressed in U2OS cells out of a total of 2193 proteins characterized by cellular heterogeneity. This study involved customizing specific antibodies for these proteins. Through large-scale immunostaining and systematic antibody specificity validation, single-cell proteomic data were obtained, unveiling a total of 539 cell cycle-dependent proteins, with 301 proteins previously unassociated with the cell cycle, constituting 56% of the discoveries (Fig. 4a [91]). This groundbreaking study established precise temporal expression profiles, tagged numerous proteins that play pivotal roles in proliferation, and marked the first temporal and spatial mapping of human proteome heterogeneity, systematically identifying cell cycle-associated proteins with heterogeneous expression at both mRNA and protein levels.

#### Spatiotemporal-based STP

The authentic STP involved the in vivo labeling of target proteins within distinct subcellular organelles using biotin in live cells, enabling the monitoring of their temporal and spatial dynamics. A recent study by Tian’s research team introduced two highly selective proximity labeling proteomics techniques [90]. These approaches revealed the spatiotemporal dynamics of interacting proteomes with remarkable temporal resolution at the subcellular level [90]. Specifically, a set of unique biotin analog probes was engineered to modulate the labeling efficiency of APEX2, thereby finely tuning the selectivity of protein complex labeling within live cells. This investigation involved the design of twelve APEX2 substrate probes, each incorporating varying electron-donating and electron-withdrawing groups strategically positioned around the phenolic structure of biotinol. These probes enable the capture of transient and weakly interacting protein complexes within living cells. A comprehensive series of in vitro and in vivo tests aimed at assessing labeling efficiency and selectivity culminated in the identification of two novel biotin analog probes, BP5 and BN2. Notably, both BP5 and BN2 probes demonstrated superior reactivity and selectivity for in vitro protein complex labeling within live cells compared to conventional biotinophenol probes (Fig. 4b [90]). This enhanced specificity in protein complex labeling is primarily attributed to their reactivity and pronounced capacity to form dimers, trimers, and even multimers, thus enabling efficient labeling of intricate protein assemblages within a confined spatial domain while reducing self-quenching. This technological advancement not only promises enhanced accuracy in deciphering protein complexes within live cells but also furnishes robust tools and methodologies for proteomic inquiries, especially in exploring intricate biological mechanisms governed by spatiotemporal dynamics at a detailed level.

### STM

Genomics typically predicts potential outcomes, proteomics elucidates ongoing processes, and metabolomics reveals past events, thereby underscoring the profound capability of metabolomics to directly and precisely characterize the terminal state and phenotype of organisms [120, 121]. STM, compared to spatial metabolomics and conventional bulk metabolomics, is particularly notable in the realms of aging and regeneration research [122, 123]. STM offers precise tracking of temporal dynamics, enabling the pinpointing of transient regenerative events and distinguishing age-related metabolic changes within specific cellular compartments. This enhances our understanding of aging and regeneration with a finer level of detail. Notably, the biosynthesis, accumulation, and catabolism of metabolites in organisms exhibit a highly precise spatiotemporal distribution. The physiological functionalities of organisms are intricately intertwined with the spatial distribution of metabolites within tissues, extending even to the level of individual cells [24, 124]. Therefore, unraveling metabolite heterogeneity in both temporal and spatial dimensions is pivotal in comprehending the intricate physiological and pathological alterations occurring in organisms. Consequently, the emergence of spatial metabolomics, integrated with advanced imaging techniques, has provided a preliminary means to visualize metabolites in biological specimens, addressing our quest for metabolite visualization since the onset of the current century [125, 126]. Contemporary STM primarily relies on spatial proteomics methodologies, where tissue sections are analyzed at multiple time points, followed by the application of pseudo-spatiotemporal analysis algorithms to deduce the metabolite status across both temporal and spatial dimensions. In the following section, we will first introduce the spatial metabolomics approach, and then illustrate a representative case of STM through pseudotemporal analysis.

#### Spatial metabolomics

Spatial metabolomics, focusing on understanding the spatial distribution and organization of metabolites (small molecules involved in cellular processes) within biological systems such as organisms, tissues, or cells, employs mass spectrometry as a powerful method for the concurrent analysis of proteins, natural products, and metabolic derivatives [127–130]. Traditional mass spectroscopy techniques, lacking spatial information, have been supplemented by mass spectrometry-based imaging strategies using different ionization methods [131]. These methods include MALDI mass spectrometry [132, 133], desorption electrospray ionization (DESI) [134, 135], and secondary ion mass spectrometry (SIMS) [134, 135]. These techniques enable label-free detection and mapping of a wide array of metabolites, including small molecules, lipids, peptides, organic compounds, and elemental ions, and mapping within cells and tissues (Table 2 [123, 124, 132–145]). For instance, atmospheric pressure MALDI achieves up to 2 μm resolution [138] , Space-MALDI allows metabolic profiling at single-cell scales [137], and transmission-mode MALDI-2 (t-MALDI-2) reaches a resolution of 1 – 2 μm [138] for detecting phospholipids and certain biomolecules. DESI, operational under ambient conditions [139–141], achieves 50 – 200 μm resolution, while its variations like nanoDESI reach 10 – 15 μm [142]. SIMS-based techniques like time-of-flight (TOF)-SIMS [143] and 3D OrbiSIMS [144, 145] provide resolutions as low as 1 μm and 0.3 μm, respectively. Despite some challenges, these methods illuminate intricate molecular landscapes in tissues. Additionally, the spatial single nuclear metabolomics (SEAM) method has been introduced to tackle challenges related to segmentation and representation in SIMS data [124]. SEAM ensures the preservation of the sample’s native state through rapid and minimalistic processing, offering in situ metabolic fingerprints and individual nuclei clustering [124].

**Table 2:** Spatial metabolomics platforms

**Technique/Method**	**Tissue preparation**	**Resolution**	**Mass coverage**	**References**
MALDI	Dried sample in matrix	2 – 10 μm	0 – 20,000 Da	[132, 133]
Atmospheric pressure MALDI	Dried sample in matrix	< 2 μm	0 – 20,000 Da	[136]
Space-MALDI	Dried sample in matrix	Single-cell	> 100 metabolites/lipids per hour	[137]
t-MALDI-2	Dried sample in matrix	1 – 2 μm	0 – 20,000 Da	[138]
DESI	Solid, frozen liquid	50 – 200 μm	0 – 2000 Da	[139–141]
AFADESI	Solid, frozen liquid	300 – 500 μm	0 – 2000 Da	[123]
nanoDESI	Solid, frozen liquid	10 – 15 μm	0 – 2000 Da	[142]
SIMS	Dried samples	50 nm	0 – 1000 Da	[134, 135]
TOF-SIMS	Dried samples	1 μm	0 – 10,000 Da	[143]
3D OrbiSIMS	Dried samples	0.3 μm	0 – 1000 Da	[144, 145]
SEAM	Preserves native state of samples	1.5 μm	0 – 2000 Da	[124]

*MALDI* matrix-assisted laser desorption/ionization, *t-MALDI-2* transmission-mode matrix-assisted laser desorption/ionization-2, *DESI* desorption electrospray ionization, *AFADESI* air flow-assisted desorption electrospray ionization, *nanoDESI* nanospray desorption electrospray ionization, *SIMS* secondary ion mass spectrometry, *TOF-SIMS* time-of-flight secondary ion mass spectrometry, *3D* three-dimensional, *SEAM* spatial single nuclear metabolomics

#### Pseudotemporal-based STM

**Fig. 5:**
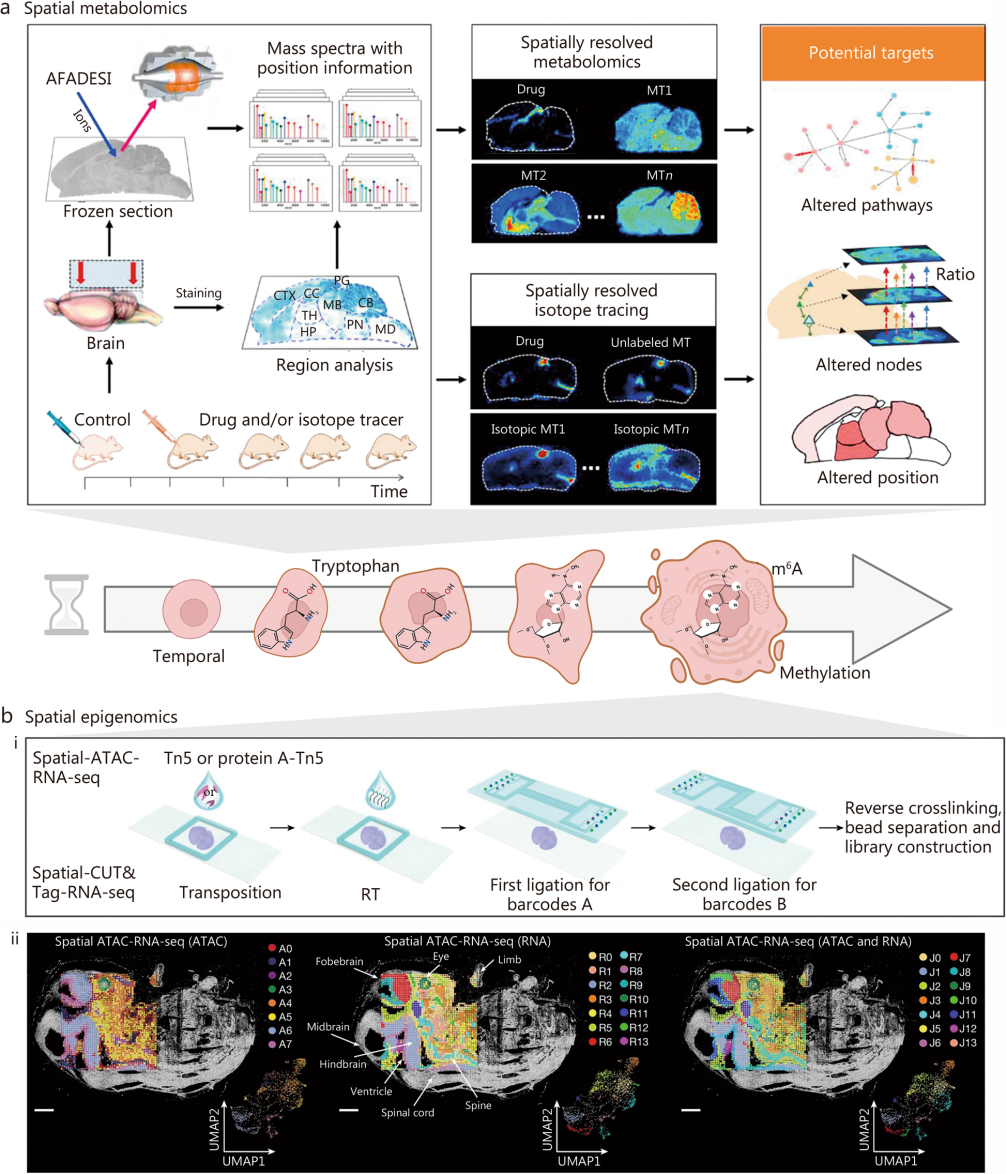
Temporal pseudotemporal analysis of spatial metabolomics and epigenomics. **a** Spatial metabolomics analyses at different time points: this section discusses an integrated approach using mass spectrometry imaging (MSI)-based spatiotemporally resolved metabolomics combined with isotope tracing to elucidate the multifaceted targets of central nervous system drugs. The analysis spans various brain regions, including the metabolite (MT), thalamus (TH), pineal gland (PG), hypothalamus (HP), midbrain (MB), cerebellum (CB), cortex (CTX), corpus callosum (CC), pons (PN), and medulla (MD). Reprinted with permission from [123]. Copyright © 2022, Chinese Pharmaceutical Association and Institute of Materia Medica, Chinese Academy of Medical Sciences. Production and hosting by Elsevier B.V. **b** Spatial epigenomic analyses at different time points. (i) Schematic workflow: this part delineates a structured workflow for spatial epigenomic analysis. (ii) Spatial distribution and uniform manifold approximation and projection (UMAP) analysis: the spatial distribution and UMAP of all clusters are presented for assay for transposase-accessible chromatin (ATAC), RNA, and ATAC and RNA data. The alignment of these clusters with tissue imaging demonstrates that the spatial clusters correspond precisely with anatomical regions. The pixel size for this analysis is set at 50 µm, with scale bars representing 1 mm. Reprinted with permission from [146]. Copyright © 2023, Published by Springer Nature. m6A mRNA modification N6-methyladenosine, Tn5 pA-Tn5 transposase 5 pre-adapter-transposase 5, RT reverse transcription, CUT&Tag cleavage under targets and tagmentation

Built upon spatial proteomics methodologies, the analyses conducted at these intervals enable a refined mapping of metabolite fluctuations, enhancing our understanding of their dynamics within biological systems. We delve into an in-depth discussion of this pseudo-STM approach, drawing from recent literature. Notably, Jin et al. [123] recently employed their novel air flow-assisted desorption electrospray ionization (AFADESI)-MSI technique, merging spatially resolved metabolomics with isotope tracer analysis. Their extensive study not only examined the action mechanism of the sedative-hypnotic drug YZG-331 but also performed a multi-target analysis using an established mass spectrometry imaging method. This investigation provided a systematical exploration of both the spatial and temporal distribution of endogenous metabolites within specific microregions of the rat brain following YZG-331 administration, showcasing the sensitive and comprehensive capabilities of AFADESI-MSI (Fig. 5a [123]). The study identified functionally relevant metabolites associated with the drug action of YZG-331, localizing them within two metabolic pathways. Analysis of the “glutamine-glutamate-gamma aminobutyric acid (GABA)” metabolic pathway and isotopic glucose tracer analysis indicated a significant increase in the GABA/glutamate ratio in the hypothalamus, suggesting an enhanced glutamate decarboxylase activity post-administration of YZG-331. Furthermore, examining the “histidine-histamine-1-methylhistamine” metabolic pathway, coupled with isotopic histamine tracer studies, mainly attributed to increased peripheral histamine penetrating the pineal gland. Additionally, 1-methylhistamine levels significantly increased in the thalamic and hypothalamic regions post-administration.

This research contributes vital insights into the metabolic mechanisms underlying YZG-331’s effects, potentially informing our understanding of age-related changes and regeneration processes. Further explorations in these areas could unveil novel therapeutic interventions for aging and regenerative medicine. Moreover, this study highlights the efficacy of advanced mass spectrometry-based imaging techniques, such as AFADESI-MSI, in elucidating complex biological phenomena. The ability to map endogenous metabolites both spatially and temporally within specific brain microregions enhances our understanding of drug actions. Additionally, integrating isotope tracer analysis amplifies our capacity to unravel intricate metabolic pathways, showcasing the potential of these cutting-edge analytical techniques in aging and regeneration research.

### STE

Epigenetics, which refers to alterations in gene expression levels stemming from modifications in non-genetic sequences, is a subject of considerable focus [147]. Traditional epigenetic methods have predominantly utilized bulk sequencing, where cells from the acquired tissue are pooled before sequencing. These methods reflect an averaged state of all cells and may obscure the inherent heterogeneity among different cell types [148–150].

Technological advancements have facilitated single-cell-level epigenetic analysis, enabling researchers to isolate individual cells from the same tissue and assess their epigenetic status through sequencing [151–154]. However, this approach has its limitations, as tissue dissociation during single-cell suspension preparation may induce changes in intracellular gene expression and result in the loss of critical spatial information necessary for understanding cell function and corresponding biological mechanisms [153, 155]. To address these challenges, spatial epigenomics has emerged, encompassing two primary analysis methodologies: one based on next-generation sequencing, exemplified by the spatial cleavage under targets and tagmentation (CUT&Tag) technology [156, 157], which is the main focus of this paper; the other relying on high-resolution imaging techniques, such as multifluorescence in situ hybridization. Presently, spatial epigenomics primarily concentrates on transcriptome and proteome analysis [158], allowing for the simultaneous evaluation of spatial transcriptome and proteome profiles in the same tissue. The spatial CUT&Tag technology integrates microfluidic coding, a fundamental principle of the platform, with the CUT&Tag technology to investigate epigenetic modifications, enabling the precise localization of histone modifications. Understanding phenotypes relies on information from coupled genomic and transcriptomic analyses, but this does not tackle why identical DNA sequences exhibit diverse expression patterns in distinct cells. Integrating single-cell epigenomic analysis with transcriptomics can directly elucidate DNA epigenetic features, including DNA methylation, chromatin accessibility, and histone modifications associated with the originating transcriptomes. Current STE heavily relies on the spatial epigenomics approach, where tissue sections are examined at multiple time points. Pseudotemporal analysis algorithms are then applied to infer the status of gene epigenetic modifications in both temporal and spatial dimensions [159]. Therefore, we will begin by discussing the three frequently encountered forms of epigenetic modification: DNA methylation [160–163], chromatin accessibility [164, 165], and histone modifications [156, 166–172]. We will delineate the distinct characteristics of each modification and summarize the commonly employed methods for their investigation **(**Table 3 [156, 160–172]**)**. Subsequently, a representative case study of STE research through pseudotemporal analysis will be illustrated.

**Table 3:** Spatial epigenomics platforms

**Epigenetic modification type**	**Method/Technique**	**Characteristics**	**References**
DNA methylation	RRBS	Focuses on CpG-content regions;	[160]
		More cost-effective	
	WGBS	Provides higher coverage of CpG islands;	[161]
		Employs post-sulfite adapter labeling	
	scMT-seq method	Derived from GT-seq and scBS-seq;	[162]
		Combines DNA methylation analysis with transcriptional data from Smart-seq2	
	scMT-seq method	Separates cytoplasm (mRNA for scRNA-seq) from nuclei (DNA for methylome analysis by scRRBS)	[163]
Chromatin accessibility	ATAC-seq	Utilizes Tn5 transposase to fragment open chromatin;	[164, 165]
		Pinpoints open chromatin state regions, correlating with limited cellular transcripts;	
		Simplified & efficient; Avoids extraction or enzymatic digestion;	
		This category involves post-translational modifications of histones affecting gene expression; Specific techniques and details were not provided in the initial passage, so this section is a general representation of the modification	
Histone modifications	ChIP-seq	Studying protein interactions with DNA	[156]
	CUT&RUN	Investigating histone modifications	[166]
	CUT&Tag	Uses Tn5 transposases to label antibody binding sites	[167]
	scCUT&Tag	Refinement for single-cell level analysis, targeting active regulatory elements through antibody-directed labeling. Specifically, targets domains bound by RNA polymerase II	[168]
	scCUT&Tag2 for1	Further refinement of scCUT&Tag, targets domains bound by Polycomb repressive complexes	[169]
	sccut-Tag-pro	Analyzing protein-DNA interactions and surface protein abundance at the single-cell level	[170]
	pair-Tag	Combining RNA and histone modification characterization; Uses combinatorial barcoding for high-throughput detection of transcriptome and chromatin occupancy	[173]
	Co-TECH	Investigates RNA and histone modifications; Like pair-tag, it utilizes combinatorial barcoding for the simultaneous high-throughput detection of the transcriptome and chromatin’s binding proteins	[146]

*RRBS* Reduced representation bisulfite sequencing, *WGBS* Whole genome bisulfite sequencing, *CUT&Tag* Cleavage under targets and tagmentation, *ATAC-seq* Assay for transposase-accessible chromatin sequencing, *GT-seq* Genome and transcriptome sequencing, *CoTECH* Combinatorial barcoding method allowing high-throughput single-cell joint detection of chromatin occupancy and transcriptome, *ChIP-seq* Chromatin immunoprecipitation followed by sequencing, *scCUT&Tag pro* a multimodal assay for profiling protein–DNA interactions coupled with the abundance of surface proteins in single cells

These three epigenetic modifications intricately influence genomic function and exhibit cell type specificity. Simultaneously, there is a profound correlation between tissue structure and cellular function. As such, spatial epigenomics holds the potential to inaugurate a groundbreaking era within the field of advanced spatial genomics. In early 2022, Deng et al. [157] utilized the spatial CUT&Tag technology, an in-situ tissue encoding method, to pioneer a high spatial resolution analysis targeting specific histone modifications. This breakthrough facilitates a comprehensive examination of tissue development across spatial and genome-wide dimensions, elucidating epigenetic mechanisms underpinning both development and disease. Thereafter, Deng et al. [173] utilized spatial ATAC-seq technology to achieve in situ spatially resolved whole-genome sequencing of chromatin accessibility within tissues, marking a significant advancement. They further delved into epigenetic regulation by introducing combined spatial epigenome and spatial transcriptome sequencing approaches, embracing spatial multi-omics technologies [146]. Liu et al. [174] conducted spatial ATAC-RNA-seq combined with spatial CUT&Tag-RNA-seq analysis on mouse embryos, effectively distinguishing various organs within the embryos through the integration of epigenomic and transcriptomic data. This investigation focused on unraveling the differentiation trajectory from radial glia to postmitotic premature neurons, aiming to explore the intricate spatial and temporal correlations between chromatin accessibility and gene expression during embryonic development (Fig. 5b [146]). These findings underscore the potential of spatial ATAC-RNA-seq technology as a powerful tool for investigating gene regulatory mechanisms and unraveling spatiotemporal dynamics in the context of tissue development. Integration with single-cell data demonstrated the technique’s ability to attain cellular or near single-cell resolution.

## Spatiotemporal multi-omics techniques in aging research

In this section, we delve into the applications of spatiotemporal multi-omics in the realm of aging research, underscoring their pivotal role in unraveling the complexities of aging. This exploration includes how STT reveals aging signatures and the dynamics of cellular senescence, how STP sheds light on protein biomarkers and interaction networks relevant to aging, how STM uncovers metabolic shifts and their correlations with age-related phenotypes, and how STE decodes DNA methylation patterns and epigenetic clocks that gauge biological age. Through the integration of these multidimensional omics datasets, researchers are positioned to gain deeper insights into the molecular foundations of aging, identify novel therapeutic targets for age-related diseases, and ultimately pave the way for interventions that promote healthy aging. The convergence of spatiotemporal multi-omics with aging research signals an exciting epoch of discovery. It brings us closer to understanding the intricate tapestry of the aging process and fuels the pursuit of extending the quality and duration of human life.

### Application of STT in characterizing age-related tissue changes

**Fig. 6:**
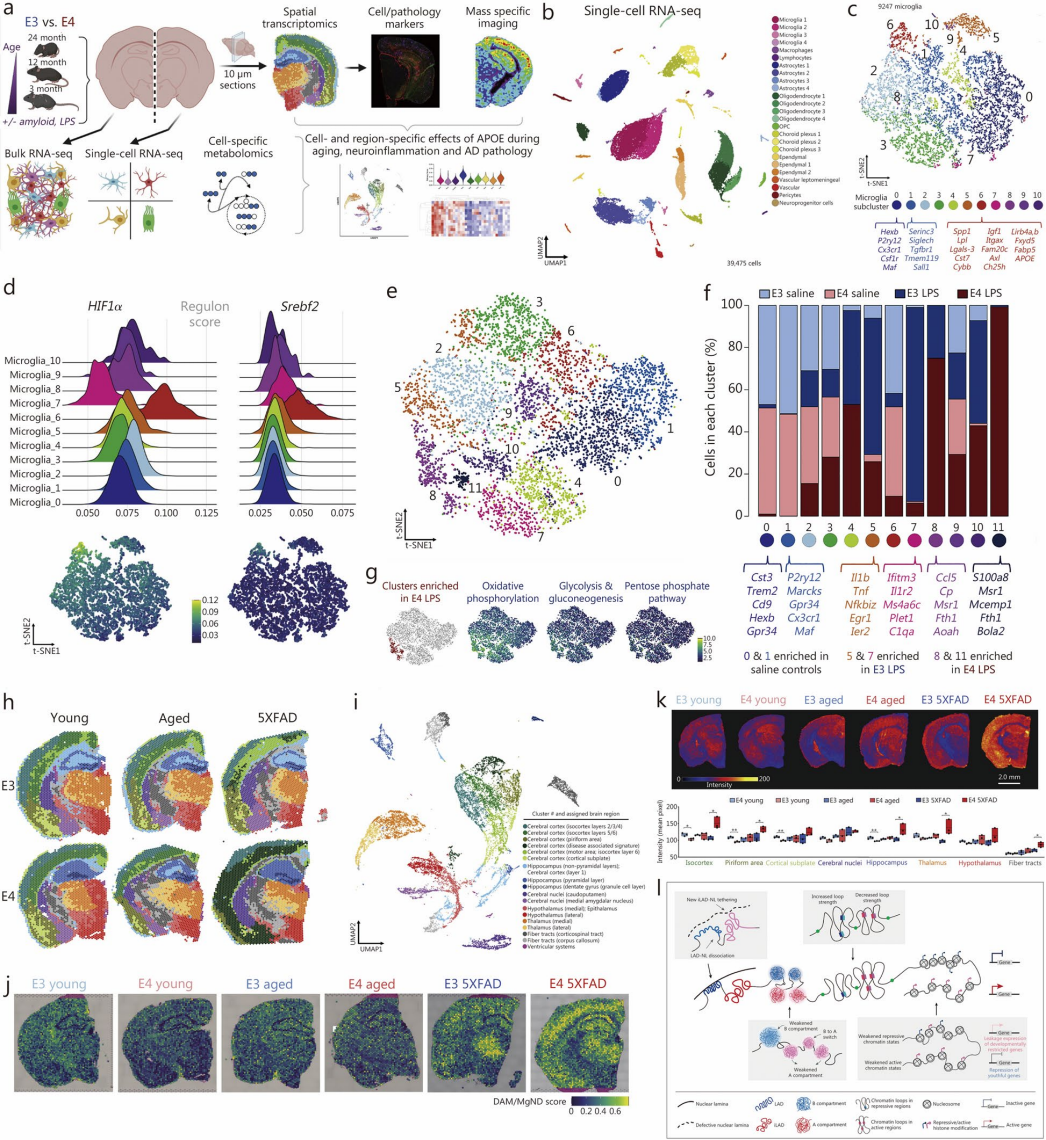
Implementing spatiotemporal multi-omics in aging research. **a** Brain analysis in APOE3 and APOE4 mice were systematically analyzed at different life stages (3, 12, and 24 months) and under specific conditions such as inflammatory challenge [lipopolysaccharide (LPS)] and Alzheimer’s disease (AD) pathology (characterized by amyloid overexpression). **b** Uniform manifold approximation and projection (UMAP) classification of gene expression clusters: a UMAP analysis identified 24 distinct clusters based on canonical gene expression markers. **c** Microglia sub-cluster analysis using t-distributed stochastic neighbor embedding (t-SNE): t-SNE revealed the microglial sub-clusters. Key biomarkers for “homeostatic” clusters (0 and 1) and disease-associated microglia (DAM)-like cluster 6 are annotated below the respective cluster labels. **d** Analysis of regulon activity scores: ridge plots (left) and t-SNE plots (right) illustrate the activity scores of *HIF1a* and *Srebf2* regulons. **e** Microglial sub-cluster distribution in LPS- or saline-treated E3 and E4 mice: a t-SNE plot displays the distribution of 12 microglia sub-clusters in mice treated with LPS or saline, differentiated by colors. **f** The allocation of experimental groups across various microglia sub-clusters is depicted through a stacked bar chart. This visualization effectively illustrates the distribution of experimental groups among the different sub-clusters of microglia. **g** Within E4 LPS brains, t-SNE plots have uncovered a significant upregulation in crucial carbon pathways related to energy production. This increased activity is notably prevalent in specific subclusters found in brains with the E4 genotype that have undergone LPS treatment. **h** Spatial transcriptomic analysis is applied to brain sections from young, aged, and amyloid-overexpressing E3 and E4 mice, offering a detailed examination of genetic expression across different brain regions and conditions. **i** A thorough UMAP analysis is conducted for comprehensive data interpretation. **j** In spatial transcriptomic plots, DAM/neurodegenerative microglia (MgND) scores are displayed for each spot, calculated via AUCell. This analysis incorporates a brain from each experimental group for comparison. **k** The study also includes an analysis of a specific lipid, phosphatidylcholine (16:0/18:2). Top scans illustrate the lipid’s spatial distribution in coronal brain sections, while the bottom part quantifies the average pixel intensity of this phosphatidylcholine variant across different brain regions, offering a visual and quantitative insight into its distribution. Reprinted with permission from [177]. Copyright © 2023 The Author(s). Published by Elsevier Inc. **l** Schematic overview of spatial genome architectures in growing (center) and senescent (gray boxes) stem cells. Reprinted with permission from [187]. Copyright © 2022, Oxford University Press. APOE apolipoprotein E, LAD-NL lamina-associated domain-nuclear lamina, iLAD inter-LAD-NL

STT enables a more comprehensive investigation of cellular interactions within the aging tissue microenvironment, facilitating the discernment of dynamic changes in gene expression patterns over time and across various spatial regions within tissues. This approach provides insights into how cells communicate and adapt in the context of aging, revealing the complex molecular mechanisms driving age-related alterations in tissue structure and function [175]. STT in aging research primarily employs the pseudotemporal-based approach, which involves sequencing tissues organized at various time points using spatial transcriptomics techniques. For instance, Hahn et al. [176] employed spatial transcriptomics combined with single-cell sequencing to map the spatiotemporal transcriptome of the aging mouse brain comprehensively. Their detailed investigation revealed pronounced regional disparities in glial cell senescence, particularly within cerebral white matter glial cells, and identified specific cerebral regions responsive to regenerative interventions. Another study employed spatial multi-omics combined with single-cell sequencing across different age points to investigate the impact of apolipoprotein E (APOE) genotypes on aging, inflammatory responses, and amyloid reactions [177]. This study highlighted the role of the microglial subpopulation (Mi_6) in APOE4 carriers and senescent AD model groups, revealing how APOE4-associated microglia promote inflammation through regulatory pathways, leading to chronic neuroinflammation (Fig. 6a-g). Second, spatial transcriptomics, revealed the prevalence of PIG^high^/OLIG^low^ in APOE4 brains, suggesting complement activation, aberrant synaptic pruning, and disrupted axonal myelin sheath formation, which perpetuates neuroinflammation and hinders lipid metabolism (Fig. 6h-j). Furthermore, spatial metabolomics techniques identified a region in APOE4 brains associated with lipid metabolism (Fig. 6k), elucidating regulatory mechanisms making certain brain regions more susceptible to neurodegeneration in APOE4 carriers. Other studies, such as those by Stoeger et al. [178] have investigated the molecular aspects of aging by analyzing transcriptomic data from multiple studies, finding that changes in transcript length are associated with longevity. Spatial transcriptomics has been instrumental in uncovering the complex mechanisms underlying age-related changes in various tissues. Russ et al. [179] utilized this technique to examine transcriptomic changes in young and aged mouse ovaries, identifying cell-specific mechanisms that contribute to age-related fertility decline. Building on this approach, Ståhl et al. [19] mapped gene expression patterns in aged brain tissue at different times to reveal spatially distinct changes associated with aging, providing insights into the temporal dynamics of gene expression. Further demonstrating the utility of spatial transcriptomics, Asp et al. [44] characterized age-related heterogeneity within tissues, showing how different cells respond to aging processes. Besides, Kiss et al. [180] employed spatial transcriptomics to pinpoint regions in the aging mouse where senescent cells accumulate, leading to the development of inflammatory foci. This accumulation may impact age-related cognitive decline and dementia, linking cellular senescence to specific pathological outcomes in aging brains. Additionally, the utilization of spatial transcriptomic techniques and statistical methods has significantly advanced our understanding of spatial gene expression patterns, cellular senescence, and age-related processes. The introduction of Giotto by Dries et al. [181] marked a significant enhancement in analyzing and visualizing spatial transcriptomic data through a comprehensive, flexible, robust, and open-source pipeline. This development set the stage for further innovations, such as SPARK by Sun et al. [182], a statistical method specially designed to identify spatial expression patterns in spatially resolved transcriptomic, advancing our capability to interpret complex data landscapes. Building on these analytical advancements, Zhao et al. [183] introduced BayesSpace, a Bayesian method that not only enhances resolution in spatial transcriptomic data but also facilitates detailed clustering analysis, allowing for finer distinctions in tissue sample studies. Concurrently, Shang et al. [184] developed SpatialPCA, which extracts low-dimensional representations of spatial transcriptomics data while preserving the inherent biological signals and spatial correlations — essential for understanding cellular senescence and spatial gene expression patterns. Further complementing these methodological innovations, LaRocca et al. [185] discovered that noncoding repetitive element transcripts accumulate with age, serving as a reliable marker of biological age. Lastly, Kasemeier-Kulesa et al. [186] bridged single-cell and spatial transcriptomics through age- and location-matched scRNA-seq and 10× Genomics Visium analyses, providing a comprehensive view of gene expression and cellular behavior in aging tissues.

Collectively, these studies provide valuable insights into the spatial distribution of gene expression, the impact of cellular senescence on cognitive decline and neuroinflammation, and the potential mechanisms underlying regenerative and non-regenerative healing. The development of robust pipelines and statistical methods has enabled more effective analysis and visualization of spatial transcriptomic data, facilitating a deeper understanding of cellular processes and interactions within tissues. These findings have significant implications for future research in understanding the molecular changes underlying aging and developing novel therapeutic strategies for age-related disorders.

### STP and STM approaches to elucidate molecular dynamics during aging

STP has revolutionized our understanding of aging by providing a dynamic perspective, enabling the identification of key specific proteins and pathways central to age-related changes [188]. This knowledge is invaluable for developing interventions targeting the molecular drivers of aging. A recent study employing STP systematically analyzed comprehensive proteomic profiles across various brain regions in non-human primates from fetuses to neonates [189]. This approach offers valuable insights into normal brain development and informs our understanding of mechanisms underlying dysfunctions and disorders in humans, with profound implications for neuroscience research. Hosp et al. [190] utilized STP to investigate proteomic changes during the progression of Huntington’s disease (HD) in the R6/2 mouse model. This study highlighted substantial reconfiguration of the soluble brain proteome, closely associated with the emergence of insoluble aggregates as the disease advanced. The accumulation of proteins in these aggregates was linked to their expression levels and specific sequence characteristics, underscoring the role of cellular protein dysfunction in HD toxicity. STP has identified proteins associated with aging, such as telomerase [191, 192] and sirtuins [193, 194]. The aberrant modulation of protein expression and functionality during aging is recognized as a key influence on the aging phenomenon [195]. Telomerase, typically quiescent in most adult cells but active in cancer cells and stem cells, presents a promising target for potentially decelerating or even reversing the aging process [191]. Huang et al. [196] utilized spatial proteomic analyses in 4 distinct brain regions, revealing differential proteins associated with resilient populations in AD. These studies underscore the significance of STP in elucidating key protein targets in aging and forming a basis for therapeutic interventions in age-related diseases.

Similarly, STM provides a comprehensive view of metabolic changes throughout the aging process. Walker et al. [197] at the University of California produced the first spatial and temporal metabolite map of the mouse brain across the aging spectrum, from adolescence to old age. This extensive dataset, encompassing 1547 molecules across 10 brain regions and 4 developmental stages [197], highlights significant variations in metabolites with no sex correlation. Notable findings include changes in sphingolipid patterns, indicative of myelin remodeling, and metabolic pathways during aging. The study revealed weakening metabolic correlations in the cerebrum from adolescence to adulthood and reduced cerebral segregation in old age [198]. These metabolic shifts, when correlated with gene and protein brain atlases, offer insights into brain metabolism’s spatial and temporal dynamics during aging. Additionally, spatial metabolomics techniques have been employed to investigate age-related metabolic changes, revealing spatially distinct metabolic signatures of aging [199]. Integrating spatial proteomic and metabolomic data has facilitated the identification of molecular interactions and pathways involved in aging [200–202]. These studies highlight the importance of proteomics and metabolomics in comprehensively elucidating the molecular mechanisms of aging in both spatial and spatial dimensions, leveraging advancements in STP and metabolomic techniques. Understanding molecular dynamics within tissues aids in deciphering age-related changes, identifying potential biomarkers, and developing targeted interventions for healthy aging.

### STE profiling in a spatial context to understand age-related modifications

STE has arisen as a transformative methodology, enabling the elucidation of intricate interactions among epigenetic modifications, spatial arrangement, and temporal dynamics within the context of aging. This section delves into the pivotal function of STE in enhancing our comprehension of the molecular mechanisms underlying the aging process. Drawing upon perspectives from prominent researchers and recent empirical discoveries, this section insights into the diverse applications of STE in the field of aging research. STE provides a dynamic view of how our epigenome evolves with age. By understanding these changes within a spatial and temporal framework, we gain unprecedented insights into the epigenetic basis of aging, offering potential avenues for interventions to promote healthy aging.

The aging process is characterized by a multitude of epigenetic alterations, encompassing DNA methylation, chromatin accessibility, and histone modifications [2, 3, 203]. These tightly regulated and frequently reversible modifications exert influence over gene expression and various cellular processes, ultimately contributing to the onset and progression of numerous age-associated human disorders. A plethora of enzyme systems, including DNA methyltransferases, histone acetylases, deacetylases, methylases, and demethylases, in conjunction with chromatin remodeling factors, participate in establishing and maintaining epigenetic patterns [204].

The landscape of DNA methylation in humans has undergone cumulative alterations over time [204]. Initial investigations highlighted generalized age-related hypomethylation, yet subsequent analyses unveiled specific loci, including those associated with certain tumor suppressor genes and polycomb target genes, exhibiting heightened methylation levels during aging [203]. Additionally, cells derived from individuals and mice manifesting progeria-like syndromes demonstrate DNA methylation shifts that partially mirror those observed in typical aging processes [203, 205]. However, the functional implications of these age-associated DNA methylation modifications remain elusive, primarily due to a lack of understanding regarding their temporal and spatial dynamics. STE, equipped to elucidate the distribution of epigenetic marks within cells and tissues across space and time, introduces a fresh vantage into the molecular dynamics underpinning aging. Rodriguez-Muela et al. [206] pioneered the use of spatial epigenomics to investigate how DNA methylation patterns shift with age, identifying specific epigenetic alterations that vary distinctly across different tissue regions. Building on this foundation, Zocher et al. [207] extended these analyses to brain tissue, where they mapped the spatial variability of DNA methylation changes, further detailing the epigenetic landscape of aging neural tissue. Smith et al. [208] underscored the importance of comprehending these spatiotemporal dynamics, emphasizing that a comprehensive grasp of epigenetic modifications throughout the aging process is crucial for developing targeted anti-aging therapies. Complementing these findings, Wu et al. [209] broadened the scope of the STE study by examining how the common mRNA modification N^6^-methyladenosine (m^6^A) affects primate tissue health and aging. They discovered tissue-specific m^6^A changes in the liver, heart, and skeletal muscle of both young and aged nonhuman primates, particularly emphasizing the susceptibility of skeletal muscle to m^6^A reduction during aging and highlighting the crucial role of the m^6^A methyltransferase-like 3 (METTL3) in maintaining muscle health. These findings shed light on the mechanisms underlying tissue aging and reveal a METTL3-m^6^A- nephronectin (NPNT) axis that helps mitigate muscle degeneration associated with aging.

Aging is strongly associated with both global histone loss and tissue-specific alterations in post-translational modifications of histones [3, 210]. Enhanced histone expression has been linked to extended lifespan in Drosophila, while investigations in fibroblasts from older individuals and patients with progeria have revealed increased histone H4K16 acetylation or H3K4 trimethylation, alongside reduced levels of H3K9 or H3K27 trimethylation [211]. These modifications of histones have the potential to induce shifts in transcriptional activity, disrupt cellular homeostasis, and contribute to age-related metabolic decline. Notably, the diminishment of telomeric heterochromatic markers has been demonstrated to result in telomere elongation.

In addition to DNA and histone-modifying factors, various chromosomal proteins and chromatin remodeling factors, including heterochromatin protein 1a and polycomb histones, play a role in genome-stabilizing DNA repair and senescence regulation [212]. The lowest hierarchical level of chromosomal organization involves the folding of chromatin fibers, intimately associated with chromatin recycling [187]. This recycling process, characterized by myriad DNA and histone modifications, plays a pivotal role in transcriptional regulation, determining DNA accessibility to the transcriptional machinery (Fig. 6l [187]). Modifications in these epigenetic elements result in significant shifts in chromatin configuration, encompassing widespread heterochromatin depletion and repositioning, prevalent occurrences in senescent cells. For instance, Zhang et al. [213] employed advanced epigenetic high-throughput methods, including chromatin accessibility sequencing, chromatin immunoprecipitation sequencing, limited enzyme digestion, and isotope-labeled quantitative proteomics, to unveil fundamental patterns of histone epigenetic modifications in senescent cells, elucidating the dynamics of chromatin spatial accessibility, and highlighting the significant contributions of epigenetic factors like lysine demethylase 4. The study underscores the potential targeting value of these molecules for manipulating senescence.

STE offers an innovative perspective for exploring the dynamic epigenetic alterations accompanying aging. By unveiling the spatiotemporal distribution of epigenetic marks, this paradigm enhances our understanding of the molecular mechanisms driving the aging process while delineating potential avenues for interventions aimed at fostering healthy aging.

## Spatiotemporal multi-omics techniques in regeneration studies

Regeneration stands as a remarkable biological phenomenon observed across the animal kingdom, encompassing a broad spectrum of organisms, from invertebrates like planarians to vertebrates like salamanders. However, within mammals, including humans, regenerative potential remains comparatively limited, predominantly confined to select tissues, such as the liver and skin. In the realm of regenerative medicine, the integration of spatiotemporal multi-omics approaches holds great promise. Drawing upon insights gleaned from existing literature, the hallmarks of regeneration can be conveniently categorized into three principal domains: the classification of regeneration types, the underlying mechanisms governing regeneration, and the regulatory processes that orchestrate these events. Regeneration is typically classified into three types, complete, incomplete, or compensatory, depending on the extent of restoration achieved [214–217]. Complete regeneration entails the comprehensive reinstatement of a lost or damaged component, achieving both structural and functional recovery. This phenomenon is observed in organisms as diverse as planarians. In contrast, incomplete regeneration results in only partial restoration, potentially recovering some, but not all, aspects of structure or function, as seen in peripheral nerves. Compensatory regeneration, on the other hand, allows an organ or tissue to regrow to functional sufficiency without replicating the original form or achieving complete functionality. A prime example is live regeneration in various vertebrates, including humans, where hepatocytes proliferate to regain function, though not the original liver structure (Fig. 1b).

The processes governing regeneration predominantly encompass cell proliferation, migration, differentiation, and the precise patterning of regenerating tissues and organs [218, 219]. Cell proliferation serves as the foundational process, where new tissues or organs arise through cellular division and propagation [220–223]. Cell migration involves the movement of cells from one location to another, establishing the correct structural arrangement [221, 224, 225]. Cell differentiation marks the transformation of cells from an undifferentiated state into specialized cell types, such as muscle or nerve cells [220, 221]. Pattern formation of regenerating organs refers to the capacity of newly generated tissues or organs to develop in a specific sequence and arrangement during the regeneration process [226, 227]. Governing the regeneration process is an array of influential factors, encompassing genes (i.e, *MSX*, *p53*, *HOX*) [228–233], environmental elements (i.e*.*, temperature) [234, 235], hormones (i.e., androgens and estrogens) [128, 129, 236–239], and growth factors (fibroblast growth factor and epidermal growth factor) [240–243]. Of particular significance, genes play a pivotal role in the regulation of cell proliferation, differentiation, and migration, intricately controlling the complex process of regeneration.

The integration of spatiotemporal multi-omics techniques into regenerative medicine holds the potential to offer invaluable insights into the intricate and dynamic molecular events governing tissue regeneration. This integration may pave the way for novel therapeutic approaches and enhance our understanding of the regenerative potential within mammals, including humans. In this review, we explore recent applications of spatiotemporal multi-omics approaches within the field of regenerative medicine, shedding light on their potential to revolutionize our understanding of regenerative processes and their therapeutic implications.

### Utilizing STT in investigating tissue regeneration processes

**Fig. 7:**
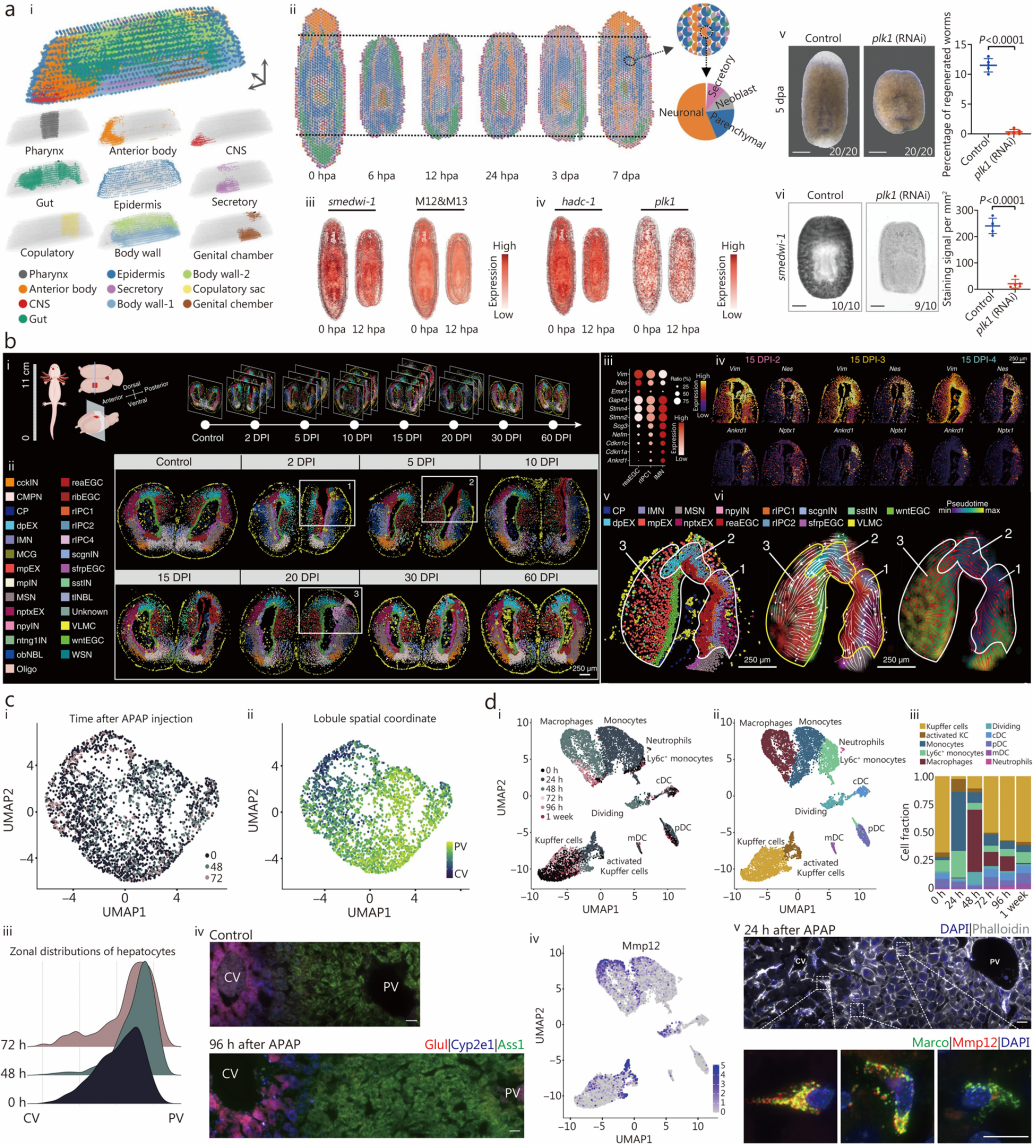
Leveraging spatiotemporal multi-omics in regenerative studies. **a** Planarian regeneration analysis: (i) spatial transcriptomics spots were grouped using the STAGATE method, labeling clusters based on their spatial expression patterns. (ii) The study visually tracks primary cell types’ spatial distribution during regeneration stages using pie charts in corresponding spatial transcriptomics spots. (iii) Spatial distribution mapping of *smedwi-1* and neoblast-related genes provides insights into their localization. (iv) Spatial transcriptomics data at 0 and 12 hpa examines spatial expression patterns of regulatory genes *hdac-1* and *plk1* during early regeneration. (v) A *plk1* knockdown effect in planarians at 5 dpa is illustrated in a bright-field image. (vi) WISH staining of *smedwi-1* in control and *plk1* knockdown planarians at 3 dpa. Reprinted with permission from [245]. Copyright © 2023 The Author(s). Published by Springer Nature. **b** Axolotl telencephalon regeneration research: (i) schematic illustrates sample collection and examined sections during homeostatic and regenerative phases. (ii) Stereo-seq maps cell types in axolotl telencephalon sections using various stages of regeneration. (iii) Bubble chart displays the fluctuating expression of marker genes defining cell types crucial for telencephalon regeneration. (iv) Heatmap shows spatial expression trends of essential markers within the injured area at 15 DPI-2, -3, and -4. (v) The spatial arrangement of cell types around the regeneration site is shown for the 15 DPI-4 section. (vi) RNA velocity plots illustrate predicted cellular lineage transitions in regenerating axolotl telencephalon area. Reprinted with permission from [60]. Copyright © 2022, The American Association for the Advancement of Science. **c** Hepatocyte study post-APAP injection: (i) UMAP visual representation of hepatocytes color-coded by time post-APAP injection. (ii) UMAP of hepatocytes color-coded by inferred spatial coordinates within the lobule. (iii) Distribution of spatial coordinates among hepatocytes for different time intervals post-injection. (iv) smFISH images of a liver lobule exhibit zonated genes at different time points post-APAP. Reprinted with permission from [246]. Copyright © 2022 Elsevier Inc. **d** Myeloid cell analysis post-APAP administration: (i) UMAP visualization of myeloid cell populations color-coded by time post-APAP administration. (ii) UMAP color-coded by myeloid cell types. (iii) Proportions of various myeloid cell subtypes at each examined time point. (iv) UMAP visualization of myeloid cells, colored by expression levels of Mmp12. (v) smFISH scan (top) and zoomed-in insets (bottom) of a liver lobule 24 h after APAP injection. Reprinted with permission from [246]. Copyright © 2022 Elsevier Inc. CNS central nervous system, hpa hours post-amputation, dpa days post-amputation, WISH whole-mount in situ hybridization, reaEGC reactive ependymoma cell, IMN immature neuron, rIPC1 regeneration intermediate progenitor cells 1, UMAP uniform manifold approximation and projection, APAP acetaminophen, CV central vein, PV portal vein, smFISH single-molecule fluorescence in situ hybridization, KC kupffer cells, pDC plasmacytoid dendritic cells, cDC conventional dendritic cells, mDC myeloid dendritic cells, Mmp12 matrix metallopeptidase 12, UMI unique molecular identifier

STT, integrating high-throughput gene expression profiling with spatial and temporal data, enables detailed characterization of gene expression patterns in regenerating tissues. This technique captures the spatial organization of gene expression over time, allowing researchers to identify specific cell types, signaling pathways, and molecular signatures crucial to tissue regeneration [19, 244]. It provides a comprehensive perspective on transcriptional changes during different stages of tissue repair, illuminating the dynamic cellular responses and regulatory networks involved in regeneration. For instance, Cui et al. [245] conducted a pioneering study on planarian regeneration. By merging spatial transcriptomics with single-cell sequencing, they created a 3D transcriptome atlas detailing characteristic cell distributions and gene expression patterns at six-time points, later expanded with two additional time points (Fig. 7ai). This study revealed a totipotent stem cell cluster and identified multiple gene expression patterns linked to planarian regeneration (Fig. 7aii). Using spatial module screening tools, the study pinpointed polar gene pairs crucial to wound-specific expression and to regions pre-/post-planarian. A key regulatory gene, *plk1*, was identified as critical in planarian formation and regeneration (Fig. 7aiii-iv), with functional analyses indicating its role as an early response element in blastocyst formation and subsequent regeneration events (Fig. 7av-vi). These insights notably enhance our comprehension of the intricate regulatory mechanism’s planarian regeneration, relevant to both specific planarian studies and broader mammalian regeneration research.

In the regeneration field, a multi-institute research team led by the Beijing Genomics Institute recently applied Stereo-seq to examine self-healing processes of brain injuries in axolotls [60]. They conducted Stereo-seq analysis on a cerebral cortex section from the salamander’s telencephalon during a 60-day post-recovery period (Fig. 7bi). This approach identified 28 distinct cell types (Fig. 7bii), revealing that morphological wound healing occurred around 30-d post-injury, with complete cell type restoration by 60-d post-injury. This study identified an additional cell type that exhibits patterns similar to those of reactive ependymoma cells (reaEGCs) and immature neurons (IMNs), and expressing markers from both categories (Fig. 7biii-iv). This cell type, termed “regeneration intermediate progenitor cells 1” (rIPC1) suggests a sequential transition of cell types (reaEGCs-rIPC1-IMNs) during regeneration. RNA velocity analysis confirmed the interconnected sequence of these cell types (Fig. 7bv-vi), providing compelling evidence for their interdependence in the regeneration process. The establishment of spatiotemporal cellular maps for salamander brain development and regeneration is crucial in understanding brain regeneration, the intricate structure of amphibian brains, and evolutionary brain structure trajectories. It also opens new avenues for clinical interventions in human tissue and organ self-repair and regeneration, while offering a valuable data repository for species evolution studies. Ben-Moshe et al*.* [246] conducted a comprehensive investigation into liver regeneration dynamics using spatial transcriptomics, single-cell sequencing, and bulk transcriptome sequencing. Their work elucidated the mechanisms underlying zonal hepatocyte regeneration and the supportive role of various region-specific non-parenchymal cells (Fig. 7c). They noted the downregulation of major histocompatibility complex class I and class II molecules, inhibiting adaptive immune activation during liver regeneration (Fig. 7d). This study severs as a comprehensive resource on the coordinated mechanisms of zonal liver regeneration, enriching our understanding of this complex process.

Collectively, these studies highlight the potential role of STT in regenerative medicine. Employing axolotls, planarians, and liver models, they provide invaluable insights into the mechanisms of tissue repair. By mapping gene expression changes within a spatial and temporal context, researchers can identify key genes, cell types, and pathways essential for successful regeneration. This methodology not only deepens our comprehension of regenerative phenomena but also offers valuable tools for advancing regenerative medicine research. STT is poised to unravel the complexities of tissue restoration and drive progress in regenerative therapies.

### STP and STM in identifying key factors and pathways in regeneration

Recent advancements in STP and STM have yielded powerful tools for dissecting the intricate processes involved in regeneration. This section delves into the invaluable contributions of these cutting-edge technologies in identifying key factors and pathways crucial for the successful regeneration of tissue and organs. By elucidating protein expression patterns and metabolic changes, researchers can unravel the spatially restricted molecular dynamics that underlie the process of tissue repair [34, 247, 248]. This innovative approach facilitates the discovery of novel biomarkers and therapeutic targets, thereby enhancing the prospects of regeneration.

STP is geared towards elucidating the spatial distribution and temporal dynamics of proteins within regenerating tissues. This approach has played a pivotal role in unraveling the molecular mechanisms that drive regeneration across diverse model organisms. In a notable study, Mallah et al. [249] conducted a spatiotemporal microproteomic analysis to explore traumatic brain injury (TBI) over a 10-d post-injury period. Their findings showcased a restoration of the brain’s protein profile to its pre-injury state within this timeframe. Intriguingly, they also identified a pronounced upregulation of proteins associated with Parkinson’s disease (PD) in the brain’s substantia nigra as early as 3 d post-injury, hinting at a potential association between TBI and PD. This investigation, employing spatiotemporal microproteomics, provides invaluable insights for regenerative medicine. By identifying specific protein markers and tracing their trajectories immediately following injury, the study sheds light on the intrinsic regenerative capacities of the brain. The suggested connection between TBI and PD underscores the need to comprehend the broader implications of traumatic injuries, a perspective crucial for shaping regenerative and neurotherapeutic strategies. Furthermore, the employment of in vitro models to validate these findings amplifies the translational significance of the study. Huang et al. [196] examined 4 distinct brain regions such as the caudate nucleus, hippocampus, inferior temporal parietal lobule, and the middle gyrus of the superior temporal gyrus in their study involving 11 healthy controls, 12 individuals demonstrating resilience to Alzheimer’s disease (RAD), and 20 patients with AD. Their results identified 33 proteins differentially expressed in the resilient group. Notably, 5 of these proteins — platelet-activating factor acetylhydrolase Isoform 1B subunit 3 (PA1B3), testican-3 (TICN3), intercellular adhesion molecule 1 (ICAM1), immunity-related GTPase Q (IRGQ), and aldehyde dehydrogenase 1 family member L1 (AL1L1) — were uniquely associated with RAD, suggesting their potential significance in the pathogenesis of AD. This study, by highlighting reduced soluble Aβ levels and pinpointing specific proteins linked to RAD, provides crucial insights that may prove instrumental for regenerative medicine research and future therapeutic interventions for AD.

Spatiotemporal protein expression profiling also offers fresh insights into peripheral nerve regeneration mechanisms and potential therapeutic targets. Bryan et al. [250] employed a reverse phase protein microarray approach to investigate the dynamic protein expression patterns during the 28-d course of peripheral nerve regeneration in a rat sciatic nerve transection injury model. The research delves into the spatiotemporal profiles of various proteins involved in nerve regeneration, including growth factors, extracellular matrix proteins, and those related to adhesion and migration. The findings reveal distinct changes in protein expression across different segments of the regenerating nerve and suggest potential implications for future research in understanding the molecular mechanisms underpinning nerve regeneration and identifying therapeutic targets.

STM, on the other hand, centers on the study of the changes in metabolite levels and fluxes within tissues during regeneration, offering insights into the metabolic processes that underpin tissue repair. A study on liver regeneration after acute damage integrated spatial metabolomics with spatially resolved scRNA-seq to elucidate the dynamics of regeneration [246]. This integrated approach unveiled the coordinated roles of different cell types, including hepatocytes, endothelial cells, hepatic stellate cells, and macrophages, in liver tissue repair. STP and STM have emerged as indispensable tools for dissecting the complexities of tissue and organ regeneration. They provide a comprehensive view of the molecular events that transpire during the repair process. Ongoing research in this domain holds great promise for regenerative medicine, as it offers insights into potential targets for therapeutic interventions aimed at enhancing tissue regeneration.

### Epigenetic regulation in spatial and temporal dimensions during tissue repair

Epigenetic modifications play a critical role in regulating gene expression and cellular identity during tissue regeneration [36, 251]. Spatial epigenetic profiling allows for the investigation of epigenetic changes within the spatial and temporal context of tissue repair [206]. By examining DNA methylation patterns and chromatin states, researchers can gain insights into the spatially defined epigenetic regulation of regeneration-associated genes and the establishment of cell fate during tissue repair. This knowledge enhances our understanding of the epigenetic mechanisms governing regeneration.

## Challenges and future prospects

**Fig. 8:**
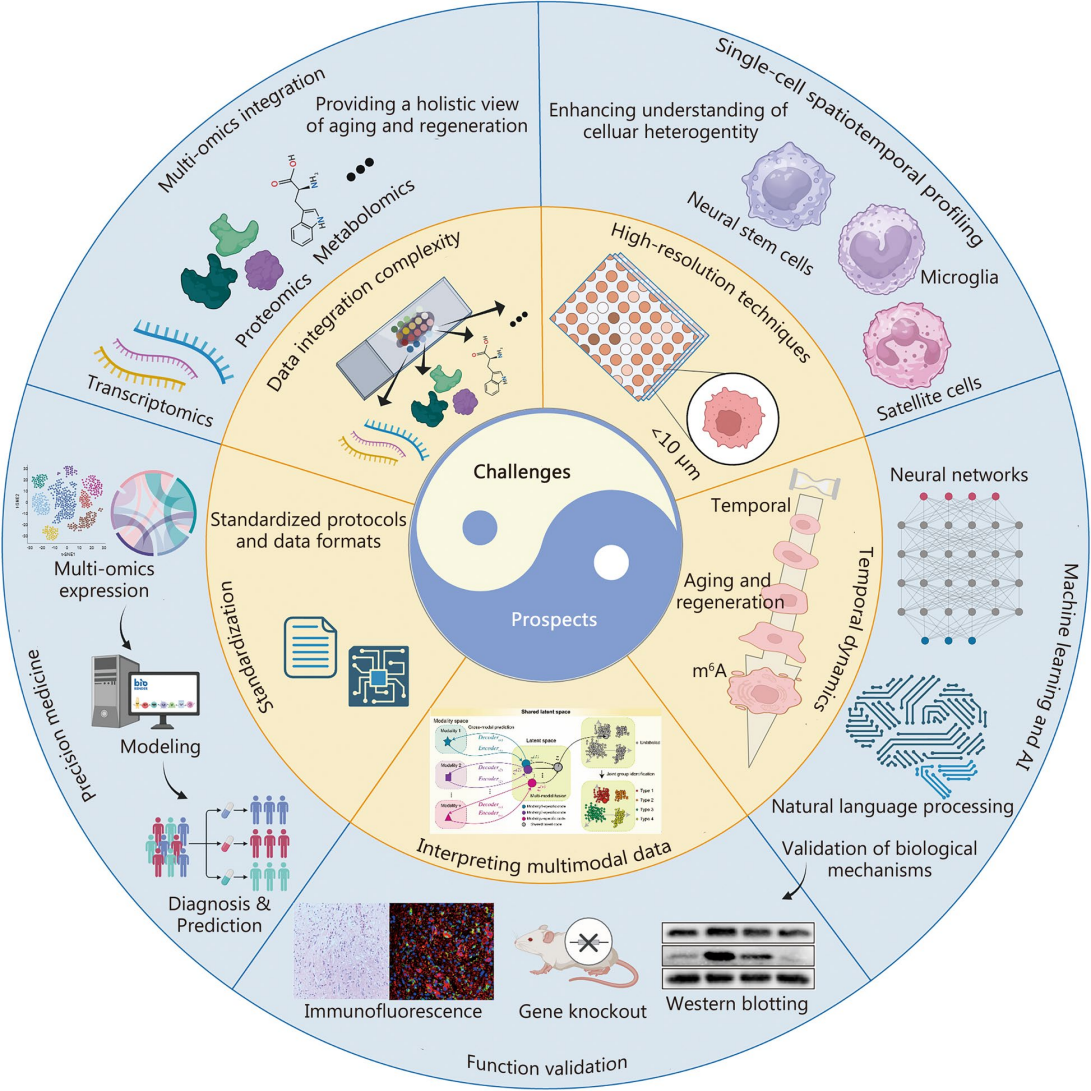
Challenges and future directions in spatiotemporal multi-omics research. In the domains of aging and regenerative medicine, challenges encompass complexities in data integration, the imperative for high-resolution techniques, comprehension of temporal dynamics, interpretation of multimodal data, and the establishment of standardization. Looking ahead, there is great promise in single-cell profiling, multi-omics integration, machine learning and AI, functional validation, and precision medicine. These advancements hold the potential to provide profound insights, interventions, and early detection capabilities in the study of aging and tissue regeneration. m^6^A mRNA modification N^6^-methyladenosine, AI artificial intelligence

In the fields of aging and regenerative medicine, a host of challenges beckon, including the complexity of data integration, the imperative for high-resolution techniques, the quest to decipher temporal dynamics, the enigma of interpreting multimodal data, and the quest for standardization. The horizon, however, gleams with promise in the realms of single-cell profiling, multi-omics integration, machine learning and AI, functional validation, and precision medicine. These avenues offer profound insights, interventions, and early detection mechanisms in the relentless pursuit of unraveling aging and tissue regeneration (Fig. 8).

### Challenges

#### Data integration complexity

In the realm of aging and regeneration research, deciphering the intricate relationship of spatial and temporal factors stands as a linchpin for uncovering the core molecular mechanisms. Traditional multi-omics approaches, while offering significant molecular insights, frequently fall short in terms of spatial and temporal resolution [82, 252–257]. This limitation threatens to obscure a comprehensive comprehension of processes like aging and tissue regeneration. Consequently, spatiotemporal multi-omics techniques that integrate both spatial and temporal dimensions have gained prominence. Such integration captures the dynamic intra-tissue changes over time and discerns molecular signatures specific to various aging or regeneration phases, enriching our understanding of spatial and temporal nuances [37, 258].

However, the analysis of extensive spatiotemporal omics datasets presents distinct challenges, necessitating bespoke computational methods. These challenges include handling the data’s high dimensionality and intricate spatial structures [259, 260], as well as discerning spatial patterns and cellular diversities [261]. In response, tools such as spatial clustering algorithms and spatially constrained dimensionality reduction have been developed. For instance, the utilization of spatially constrained non-negative matrix factorization aids in identifying unique cell types and their gene expression profiles [34, 262, 263]. Moreover, the integration of diverse spatiotemporal datasets, spanning transcriptomics to epigenomics, presents a formidable undertaking [264–266]. These datasets often derive from different platforms, leading to disparities in data types and scales. Overcoming these demands, the creation of robust analytical tools for data harmonization and the introduction of advanced techniques, including multi-omics integration algorithms, becomes imperative. These tools are instrumental in harmonizing and deciphering the varied and intricate datasets, thereby enabling researchers to distill invaluable insights from the integration of diverse omics layers. This integrative approach stands as a cornerstone for unraveling the multifaceted aspects of aging and regeneration, providing a more comprehensive understanding of their spatiotemporal intricacies. The achievement of proficient data integration thus becomes pivotal in our pursuit of comprehending the intricate spatiotemporal intricacies of aging and regeneration.

#### High-resolution techniques

Obtaining high-resolution data capable of capturing dynamic changes at the cellular and subcellular levels emerges as a categorical imperative. Existing techniques, often lacking the necessary spatial and temporal granularity to dissect intricate processes during aging and regeneration, necessitate a quantum leap. The development of cutting-edge imaging and omics technologies stands as the panacea to surmount this challenge. For instance, single-cell multi-omics approaches have the potential to unveil granular insights into individual cell behaviors over time [165, 267–269]. Advanced imaging techniques, including super-resolution microscopy and live cell imaging, offer high-resolution spatial and temporal data [270–274]. Strategic investment in research and innovation to refine and expand these techniques will be critical for gaining a deeper into the spatiotemporal dynamics within these domains.

#### Temporal dynamics

Comprehending the temporal dynamics of biological processes takes center stage in aging and regeneration research. Current methods often provide snapshots of static states, thereby rendering the capture of dynamic changes a formidable task. In Part II, spatiotemporal multi-omics bifurcate into two main classes. The first entails pseudotemporal analysis through the utilization of spatial multi-omics, examining tissue sections from diverse time intervals. The second employs genuine spatiotemporal genomics techniques, labeling specific cellular components to investigate temporal changes. However, the pseudotemporal approach remains dominant in aging research due to its established methodology and ease of use [43, 275]. Addressing this challenge requires the conception of longitudinal and time-series experimental designs. Researchers must employ technologies that facilitate real-time monitoring and sampling of biological systems over extended durations. Additionally, computational tools for modeling and analyzing temporal data necessitate enhancement to extract meaningful patterns and identify critical transition points during aging and regeneration.

#### Interpreting multimodal data

The integration of multiple omics modalities, encompassing genomics, proteomics, and metabolomics, beckons the need for comprehensive frameworks for interpreting data effectively. The intricate interactions across these layers in aging and regeneration require innovative approaches to unravel intricate networks and pathways [276]. Techniques for multimodal data integration, including network-based analyses and pathway enrichment methods, must be refined to account for dependencies and interactions between distinct omics components [277–280]. Furthermore, interdisciplinary collaboration spanning biology, bioinformatics, and systems biology, is indispensable for advancing our ability to interpret multimodal data proficiently. Contemporary biotechnology enables the simultaneous measurement of multiple high-dimensional modalities (e.g., RNA, DNA accessibility, and proteins) within a single tissue sample. To comprehensively elucidate the role of gene regulation in steering biodiversity and function, a combination of diverse analytical approaches becomes imperative. However, prevailing methods primarily cater to single-task analysis, offering only a partial insight into multimodal data. Recent breakthroughs in single-cell biotechnology empower the concurrent assessment of gene expression and other high-dimensional modalities within the same cell, yielding multimodal histological data [281–283]. This data vantage furnishes a comprehensive view of cellular transcriptional and functional processes. Nonetheless, traditional methodologies fall short of handling such multimodal data, necessitating the development of novel approaches to fully harness their potential [277, 278]. While researchers have put forth various methods for multimodal analysis, tackling tasks such as joint group identification, cross-modal prediction, and cross-modal correlation discovery, these methodologies are typically tailored with a singular objective, posing challenges for their integration into a unified framework [284–287]. To bridge this divide, Tang et al. [288] introduced interpretable multi-task deep neural networks, demonstrating efficacy in navigating the intricacies of multimodal data analysis. The network utilizes an encoder-decoder-discriminator architecture, which is iteratively trained to perform tasks such as joint cluster identification and cross-modal prediction. Such a framework holds significant potential to accelerate the uncovering of cell-type-specific regulatory dynamics across transcriptomics and other modalities.

#### Standardization

The establishment of standardized protocols and data formats for spatiotemporal multi-omics data assumes paramount importance, ensuring data reproducibility and comparability across studies [289, 290]. Presently, variations in experimental protocols, data preprocessing steps, and data reporting impede data harmonization. Collaborative endeavors among researchers are imperative to define and embrace common data standards and best practices. This includes the development of metadata standards encompassing experimental conditions, sample information, and data preprocessing steps. Moreover, the establishment of data repositories and platforms adhering to these standards becomes imperative to facilitate data sharing and reproducibility within the scientific community. Standardization efforts will enhance the reliability and utility of spatiotemporal multi-omics data in aging and regeneration research.

### Future prospects

#### Integration of additional layers such as 3D configuration and single-cell resolution

The horizon of spatiotemporal multi-omics research in the context of aging and regeneration radiates with great promise, especially within the realm of single-cell analysis. These advancements will furnish a granular perspective of individual cells as they evolve over time [291–293]. This approach will uncover previously veiled cellular heterogeneity and dynamics, enabling the discernment of rare cell populations, transitional states, and responses of cells to environmental cues. Single-cell spatiotemporal profiling is poised to be instrumental in unraveling the intricacies of tissue regeneration, elucidating the roles of distinct cell types, and meticulously tracking their behavior throughout the intricate process.

Moreover, the incorporation of additional layers of information, such as 3D configuration and single-cell resolution, constitutes a pivotal direction for advancing spatiotemporal omics within aging and regeneration research. 3D imaging techniques, including spatial transcriptomics in 3D and expansion microscopy, possess the capability to bestow spatial context with greater resolution, thereby facilitating the exploration of tissue architecture and cellular interactions [44, 294–296] . Furthermore, the fusion of single-cell transcriptomics with spatial omics techniques is poised to unveil the landscape of cell heterogeneity and lineage trajectories during aging and regeneration, ultimately streamlining the identification of critical cell types and their functional roles [24, 296–298]. Combining these multidimensional approaches will offer a more comprehensive understanding of the complex biological processes involved.

It is imperative to acknowledge that the spatiotemporal transcription approach employed by TEMPOmap may potentially introduce sequence bias, necessitating the incorporation of U analogs for metabolic labeling and thoughtful design of DNA probes [43] Subsequent investigations could synergize TEMPOmap with high-throughput single-cell functional genomics to pinpoint pivotal molecular factors shaping the kinetic landscape of the RNA life cycle. Furthermore, optimizing the conditions for metabolic labeling and integrating diverse molecular probing protocols hold the potential to extend the applicability of this method to isolated or in vivo tissue samples, thereby systematically unraveling dynamic events in tissue biology. The coordination of STT patterns may yield insights into the molecular mechanisms governing a range of biological phenomena, encompassing developmental processes, pattern formation, memory and learning, and biological rhythms, as well as the initiation and progression of disease.

#### Multi-omics integration to advance the understanding of biological systems

The future is set to witness an unwavering emphasis on the development of integrated multi-omics approaches, thereby ushering in a comprehensive and interconnected understanding of aging and regeneration processes. The fusion of genomics, proteomics, metabolomics, and other omics data will empower researchers to construct holistic models that capture the multidimensional facets of biological systems. Advanced computational tools will abet the integration of diverse omics layers, ultimately enabling the identification of key regulatory hubs and cross-talk between different molecular components. This integrated perspective is poised to pave the path toward more targeted and precise interventions and personalized therapies within the domain of aging-related diseases and regenerative medicine.

#### Addressing technical and computational challenges for broader adoption

While the realm of spatiotemporal omics teems with potential, it concurrently presents several technical and computational challenges. The formulation of standardized protocols for sample preparation, data acquisition, and analysis is essential to ensure reproducibility and comparability across studies [289, 299]. Additionally, the scrutiny of large-scale spatiotemporal omics datasets requires advanced computational methods and tools for data integration, visualization, and interpretation [280, 300]. The development of robust bioinformatic pipelines, machine learning algorithms, and statistical models assumes a crucial role in distilling meaningful insights from complex spatial omics data [288, 301–304]. Furthermore, concerted efforts should be channeled toward augmenting data sharing and fostering collaboration among researchers, thereby nurturing the progression and adoption of spatiotemporal omics within the realms of aging and regeneration.

Overall, the future trajectory of spatiotemporal omics within aging and regeneration research holds great promise. The integration of emerging technologies, the incorporation of supplementary layers of information, and the resolution of technical and computational challenges are poised to further illuminate the molecular mechanisms underlying these processes. This enlightenment will, in turn, lay the foundation for pioneering therapeutic strategies.

Machine learning and artificial intelligence (AI) are set to occupy a pivotal role in unearthing meaningful insights from the expansive and intricate datasets engendered by spatiotemporal multi-omics studies. These technologies will serve as the vanguards of data-driven discoveries by uncovering patterns, correlations, and predictive models. AI-driven analyses will help uncover novel biomarkers, pathways, and regulatory networks associated with aging and regeneration. Additionally, machine learning algorithms will facilitate data interpretation, aiding researchers in understanding the functional implications of omics findings. The integration of AI into spatiotemporal multi-omics research will accelerate our ability to uncover hidden biological mechanisms.

Machine learning and statistical modeling techniques play a crucial role in extracting meaningful insights from spatiotemporal omics data within the field of aging and regeneration. These approaches facilitate the identification of key biomarkers, regulatory networks, and predictive models. Supervised machine learning algorithms, such as support vector machines (SVMs) and random forests, can be applied to classify spatial omics data based on predefined phenotypes or outcomes. Notably, SVM has been instrumental in classifying regenerating tissue regions based on gene expression profiles, ultimately leading to the identification of genes associated with successful regeneration [305].

Unsupervised machine learning methods, including clustering and dimensionality reduction techniques, furnish the capability to unearth novel cell populations and spatial patterns within complex tissue samples. Techniques such as t-distributed stochastic neighbor embedding and uniform manifold approximation and projection have been judiciously employed to unravel cellular heterogeneity and spatial organization within the context of aging and regeneration [306, 307]. Statistical modeling approaches, inclusive of differential expression analysis and spatial enrichment analysis, aid in the identification of genes and pathways significantly associated with aging and regeneration processes. These methods open avenues for the discernment of spatially regulated genes, spatially enriched pathways, and the coordinated expression of genes in spatial domains [308, 309]. Through the adept utilization of specialized computational methods, bioinformatic tools, and machine learning techniques, researchers stand poised to glean invaluable insights into the spatial and temporal dynamics of biological processes, thereby accelerating our comprehension of aging and regeneration.

#### Emerging technologies and techniques in spatiotemporal omics

The field of spatiotemporal omics is undergoing rapid evolution, marked by the emergence of cutting-edge technologies and techniques that are poised to shape its future significantly. Notably, advancements in imaging technologies, such as multiplexed imaging and super-resolution microscopy, are catalyzing higher resolution and multiplexed analyses of spatial omics data [310–312]. Additionally, the integration of spatial transcriptomics with other omics modalities, including spatial proteomics and metabolomics, holds the potential for a more comprehensive understanding of the intricacies underlying aging and regeneration processes [313–315]. These emerging technologies herald new frontiers for exploring the multifaceted molecular dynamics in spatial and temporal dimensions.

Additionally, it is imperative to recognize that the regulatory state and functionality of cells are intricately governed by the spatiotemporal orchestration of gene expression. One pivotal contributor to the emergence of RNA expression heterogeneity lies in the precise control of mRNA metabolism and transport. To fully grasp the landscape of transcriptional and post-transcriptional gene regulatory mechanisms in cells and tissues, a systematic exploration of RNA expression across both time and space becomes imperative. However, prevailing transcriptome analysis techniques fall short of capturing the concurrent temporal and spatial attributes of RNA molecules. On one hand, current spatial transcriptome analysis methods enable a thorough dissection of gene expression within heterogeneous cell populations while considering tissue morphology. Yet, they are confined to providing static snapshots of cells and tissues, thereby failing to illuminate the dynamic flux of gene expression. On the other hand, established metabolic labeling approaches facilitate temporal profiling of nascent RNA, but they lack spatial resolution. Meanwhile, live cell imaging allows for tracking RNA trajectories within cells, yet visualizing multiple transcripts poses a formidable challenge. Consequently, there is an urgent demand for transcriptome analysis methodologies capable of affording both temporal and spatial resolution, thereby enabling the monitoring of mRNA dynamics during the life process.

#### Functional validation

While spatiotemporal multi-omics technologies provide valuable insights, the significance of functional validation studies is set to ascend. These experiments bridge the gap between omics findings and the elucidation of biological mechanisms, substantiating the relevance of identified biomolecules and pathways in the context of aging and regeneration. Functional validation encompasses experimental techniques such as gene knockout studies, perturbation assays, and functional genomics approaches [316–318]. By establishing causality and shedding light on the roles of specific molecules or pathways, functional validation studies fortify the robustness and translatability of omics discoveries, thus paving the way for targeted interventions and therapeutic development.

#### Spatiotemporal multi-omics in precision medicine

Central to the concept of aging is the notion of regeneration, denoting the body’s capacity to restore and rejuvenate compromised tissues and organs. Regenerative efficacy diminishes with age, ushering in a decline in tissue functionality and the onset of age-related ailments. Spatiotemporal multi-omics methodologies provide an unparalleled lens through which to scrutinize tissue regeneration with exceptional precision [319, 320]. By scrutinizing the spatial dispersion of cellular constituents and their molecular profiles, investigators can unveil the diversity of regenerative mechanisms across distinct tissues and life stages [321–323]. This insight proves invaluable in devising tactics to amplify tissue reparation and invigoration, potentially stalling the onset of age-linked maladies.

The assimilation of spatiotemporal insights into multi-omics analyses augments our comprehension of aging. Spatiotemporal multi-omics entails the delineation of molecular changes not solely across diverse omics strata, but also within the spatial milieu of tissues and organs. This paradigm furnishes a deeper grasp of how cellular interplays, local habitats, and cellular kinetics impact the aging trajectory [73, 300]. Furthermore, it facilitates the detection of localized molecular imprints concomitant with precise aging-associated phenotypes, proffering promising biomarkers for early detection and intervention.

The confluence of aging research, inquiries into regeneration, and spatiotemporal multi-omics paints a trajectory for precision health applications vis-à-vis aging-linked disorders. Precision medicine aims to provide tailored interventions and therapeutics grounded in an individual’s idiosyncratic genetic makeup, lifestyle, and environmental encounters [319]. Leveraging the revelations derived from spatiotemporal multi-omics, investigators can pinpoint molecular targets for interventions that foster healthy aging and mitigate the toll of age-linked maladies [324, 325]. These interventions span a spectrum from lifestyle adaptations to personalized therapies attuned to an individual’s specific molecular profile.

The interdisciplinary synergy among aging, regeneration, and spatiotemporal multi-omics promises to elevate our understanding of the aging trajectory and its associated disorders. Through untangling the intricate molecular networks governing aging, researchers are forging a path toward precision health interventions, which have the potential to extend health span and augment the quality of life for aging populations. As technology continues to advance, the quest to unlock the mysteries of aging and attain precision health becomes more auspicious than ever.

## Conclusions

Spatiotemporal multi-omics methods have emerged as invaluable tools in unraveling the complexities of aging and regeneration research. This review has elucidated their pivotal roles in illuminating molecular dynamics across both spatial and temporal dimensions. The integration of STT, STP, STM, and STE has enabled a profound exploration of age-associated tissue alterations and the intricate processes of tissue regeneration. By harmonizing spatial and temporal data, these methodologies have yielded transformative insights into the molecular underpinnings of age-related ailments and regenerative therapies. Of particular significance is the ability of spatiotemporal multi-omics to unveil the spatial heterogeneity of molecular changes associated with aging, shedding light on the fundamental cellular processes governing the aging phenotype. Spatial transcriptomics, for instance, has uncovered intricate transcriptional variations and the evolving states of cellular populations in age-related tissues. Concurrently, spatial proteomics and metabolomics have cast a spotlight on critical components within the dynamic landscape of aging, offering promising targets for therapeutic interventions.

In the realm of tissue regeneration, spatiotemporal multi-omics methods have provided intricate blueprints for regenerating tissues. They have identified distinct cell types and traced their gene expression trajectories, while also capturing the molecular and metabolic shifts that underlie successful restoration. Furthermore, studies in spatiotemporal epigenetics have enriched our understanding of tissue repair mechanisms. Looking ahead, the promise of spatiotemporal omics looms large on the horizon. Continuous technological advancements will grant us deeper and more granular insights into these biological phenomena, propelling our understanding to new heights. Moreover, fostering a culture of data sharing and collaborative efforts among researchers will solidify the indispensable role of spatiotemporal omics in the field of biomedicine.

In summary, spatiotemporal omics techniques have been transformative, offering unprecedented clarity at the molecular level and paving the way for tailored therapies and advancements in aging and regenerative medicine. The future of this burgeoning field holds immense potential to unravel the intricate mechanisms underlying aging and regeneration, ultimately leading to improved health outcomes.

## Data Availability

Not applicable.

## Abbreviations

3D: Three-dimensional
AD: Alzheimer’s disease
AI: Artificial intelligence
cDNA: Complementary DNA
CID: Coordinate encoding
DESI: Desorption electrospray ionization
m^6^A: mRNA modification N^6^-methyladenosine
MALDI: Matrix-assisted laser desorption/ionization
mRNA: Messenger RNA
PD: Parkinson’s disease
RAD: Resilience to Alzheimer’s disease
scNT-seq: Single-cell RNA tagging sequencing
scRNA-seq: Single-cell RNA sequencing
SIMS: Secondary ion mass spectrometry
STM: Spatiotemporal metabolomics
STP: Spatiotemporal proteomics
STT: Spatiotemporal transcriptomics
SVM: Support vector machine
TBI: Traumatic brain injury
s^4^U: 4-thiouridine
